# Pulmonary Toxicity of Long, Thick MWCNT and Very Long, Thin Carboxylated MWCNT Aerosols Following 28 Days Whole-Body Exposure

**DOI:** 10.3390/toxics13050401

**Published:** 2025-05-16

**Authors:** Chang Guo, Matthew D. Wright, Alison Buckley, Adam Laycock, Trine Berthing, Ulla Vogel, Frédéric Cosnier, Laurent Gaté, Martin O. Leonard, Rachel Smith

**Affiliations:** 1 Toxicology Department, UK Health Security Agency, Harwell Campus, Didcot OX11 0RQ, UK; matthew.d.wright@ukhsa.gov.uk (M.D.W.); alison.buckley@ukhsa.gov.uk (A.B.); adam.laycock@ukhsa.gov.uk (A.L.); martin.leonard@ukhsa.gov.uk (M.O.L.); 2 National Research Centre for the Working Environment, DK-2100 Copenhagen, Denmark; trb@nfa.dk (T.B.); ubv@nfa.dk (U.V.); 3 French Research and Safety Institute for the Prevention of Occupational Accidents and Diseases (INRS), Toxicology and Biomonitoring Division, 54519 Vandoeuvre les Nancy, France; frederic.cosnier@inrs.fr (F.C.); laurent.gate@inrs.fr (L.G.)

**Keywords:** nanotoxicology, carbon nanotubes, CNT, MWCNT, NM-401, rat, inhalation, whole-body, osteopontin

## Abstract

Pulmonary exposure to carbon nanotubes (CNTs) has been linked to a series of adverse respiratory effects in animal models, including inflammation, genotoxicity, fibrosis, and granuloma formation, the degree and characteristics of which are considered dependent upon the detailed physicochemical properties of the material as inhaled. To further explore the effect of variations in physicochemical properties on pulmonary effects, two different multi-walled CNTs (MWCNTs) were tested in vivo: a pristine MWCNT (pMWCNT) (NM-401) and a surface-modified MWCNT (MWCNT-COOH). Female Sprague–Dawley rats were whole-body exposed for 28 days to MWCNT aerosols (pMWCNT (0.5 and 1.5 mg/m^3^) and MWCNT-COOH (1.5 and 4.5 mg/m^3^)) and followed up to 1 year post-exposure. The inhalation exposures resulted in relatively low estimated lung deposition. Bronchoalveolar lavage fluid (BALF) analysis indicated inflammation levels broadly consistent with deposited dose levels. Lung histopathology indicated that both MWCNTs produced very limited toxicological effects; however, global mRNA expression levels in lung tissue and BALF cytokines indicated different characteristics for the two MWCNTs. For example, pMWCNT but not MWCNT-COOH exposure induced osteopontin production, suggestive of potential pre-fibrosis/fibrosis effects linked to the higher aspect ratio aerosol particles. This is of concern as brightfield and enhanced darkfield microscopy indicated the persistence of pMWCNT fibres in lung tissue.

## 1. Introduction

Carbon nanotubes (CNTs) are graphene layers in a cylindrical form being used in an increasingly wide range of applications [1]. The growing production of CNTs has increased their potential for impacting human health, especially from occupational exposure and biomedical applications, e.g., drug delivery, biosensors, and tissue engineering [2,3], and thus the need for appropriate hazard and risk assessment approaches.

Inhalation is generally considered the critical exposure pathway for CNTs [4] and in vivo studies have indicated that pulmonary exposure to CNTs is linked to a series of ad-verse respiratory effects, including oxidative stress, inflammation, and genotoxicity, as well as fibrosis and pulmonary tumours (e.g., as reviewed [4,5,6]) and pleural mesothelioma [7], although some inhalation studies have found no or very limited pulmonary effects [8,9].

One of the major challenges in nanotoxicology is to be able to predict human risk following long-term exposure to low exposure levels based on the results of short-term studies in animal models. For CNTs, it is even more complex as CNTs can differ greatly in their physicochemical properties (e.g., length, thickness, aspect ratio, shape, agglomeration state, rigidity, metal impurities, surface modifications), so, typically, hazard predictions for one form of CNT cannot be directly read across to another. Improving understanding of the impact of different physiochemical characteristics of CNTs and the aerosols they form on toxicological endpoints following inhalation can assist in the development of approaches to grouping and read across for hazard and risk assessment, and contribute to safe-by-design (SbD) approaches.

A number of in vivo studies have investigated the effect of CNT physicochemical characteristics on pulmonary endpoints by comparing two or three materials. For exam-ple, a pharyngeal aspiration study in mice found that a long, rigid, multi-walled CNT (MWCNT-7) had a more pronounced fibrotic effect than a shorter, highly agglomerated MWCNT (Nanocyl) [10]; an intratracheal instillation study in mice using long (5–15 µm) and short (350–700 nm) MWCNTs found that only the long increased collagen deposition and pulmonary fibrosis [11]; an instillation study in spontaneously hypertensive rats found that long (20–50 µm) but not short (0.5–2 µm) MWCNTs increased fibroblast pro-liferation, collagen deposition, and granuloma [12]; and Vietti et al. [13] found that the lung content of hydroxyproline (marker of collagen accumulation) in mice exposed by pharyngeal aspiration to NM-400 increased significantly in comparison to control, but that no effect was seen for a sample of crushed NM-400 or MWCNTg2400, both of which were short fibres. All the above comparative studies suggested that fibre length is an important determinant of pulmonary fibrosis development following pulmonary exposure to MWCNT.

Other comparison studies have focused on alternative characteristics and endpoints. For example, Xu et al. [14] compared the effects of straight (length 8 µm, diameter 150 nm) and tangled (fibre length 3 µm, diameter 15 nm) MWCNTs delivered by intratracheal pulmonary spraying in male F344 rats once every 2 weeks for 24 weeks (total delivered dose 1.625 mg/rat). It was found that the straight, but not the tangled MWCNT, translocated into the pleural cavity and induced fibrosis and patchy parietal mesothelial proliferation lesions and induced strong inflammatory reactions in the pleural cavity lavage. In contrast, the tangled material induced stronger inflammation in the lung tissue than the straight. The authors considered that the results suggested that the straight MWCNT had a greater capacity to cause asbestos-like pleural lesions relevant to mesothelioma development, and further, that the straight MWCNT was possibly more active in the mesothelium and the tangled MWCNT was possibly more active in the lung. In a further, longer-term study by the same group, Saleh et al. [15] compared the carcinogenicity in male F344 rats of two different MWCNTs, a straight MWCNT (fibre diameter 160 nm, length 6 ± 3 µm) and a tangled MWCNT (fibre diameter 7 nm, particle agglomerate diameter 1.0 ± 0.7 µm), delivered by intratracheal pulmonary spraying once a week over a 7 week period (8 administrations from day 1 to day 50, total doses 0.5 and 1 mg) followed by a 2 year observation period, with crocidolite asbestos (1 mg) used as a positive control. The study found no pleural mesothelioma in any group, and no significant increase in bronchiolo-alveolar hyperplasia or lung tumours for the asbestos or the straight MWCNT, but evidence of hyperplasia and adenoma and adenocarcinoma in the animals exposed to the tanged MWCNT. A number of possible hypotheses for the partially unexpected results of this second study were proposed, including the impact of the larger diameter of the straight MWCNT and the greater retention and surface area dose of the tangled MWCNT, which might perhaps have resulted in pulmonary ‘overload’ conditions. These studies serve as a useful example of the complexity of the links between physicochemical characteristics and biological effects.

Some studies have used panels of materials to investigate the effects of a range of characteristics on a number of endpoints. For example, Fraser et al. [16] investigated in vitro the impact of physicochemical characteristics of a range of MWCNT and carbon nanofibers (CF) used or produced in US facilities on a range of endpoints and found that larger tube diameters, greater lengths, and bundled agglomerate forms were associated with greater severity of effects. Their results also indicated that distributions of physical dimensions provided more consistent grouping of materials with respect to toxicity than average values alone.

Several linked studies have systematically explored the effect of a range of physico-chemical characteristics of CNTs using intratracheal instillation. Poulsen et al. [17] exposed mice to a single dose of 10 commercial MWCNTs with different dimensions and surface modifications and trace metal contents. The endpoints considered were BALF neutrophil infiltration (inflammation) and total protein, genotoxicity, and histopathology of lung tissue at post-exposure days 1, 28, and 92. Correlations between neutrophil influx and dose, surface area (BET), and some metals (Mn, Mg, Co) were identified, and diameter was a positive predictor of genotoxicity. Significant covariance between many physico-chemical parameters was, however, a limitation of the study. In a further study by the same group, mice were exposed by intratracheal instillation to a panel of 11 MWCNTs, including some used in the previous study but also additional materials, including thin and entangled and functionalized materials [18]. The endpoints assessed at 1 year were lung histopathology and genotoxicity in the liver and spleen by comet assay. Short and thin MWCNTs were observed as agglomerates in lung tissue 1 year after exposure, whereas thicker and longer MWCNTs were detected as single fibres, suggesting the biopersistence of both types of MWCNTs. The thin and entangled MWCNTs induced varying degrees of pulmonary inflammation, in terms of lymphocytic aggregates, granulomas and macrophage infiltration, whereas two thick and straight MWCNTs did not. Multiple regression analysis revealed that both a larger diameter and higher content of iron predicted fewer histopathological changes, whereas higher cobalt content significantly predicted more histopathological changes. It was hypothesized that the “protective” effect of Fe was probably caused by the negative correlation with Mn, Mg, and Co. Co content was identified as a possible predictor of lymphocytic infiltration and granuloma formation. No MWCNT-related fibrosis or tumours in the lungs or pleura were found. Another study by the same group explored whether genotoxicity and pulmonary inflammation produced by instilled MWCNTs (mean length 3.9 µm) changed if the fibres were reduced in length (1 µm) or if COOH groups were added. They found the original MWCNT induced genotoxicity, which was absent for the shorter CNT and also after the introduction of COOH groups, and that these variants also produced lower levels of inflammation [19]. More recent studies involving the same group and using a similar methodology have explored the influence of surface functionalisation and single vs. multiwalled structures on genotoxic and inflammatory responses for a panel of 12 CNTs and found MWCNTs were more genotoxic than SWCNTs [20], and also compared the inflammatory and acute phase response to 26 CNTs with a range of physicochemical properties. Using Pearson correlation analysis, two clusters of highly correlated parameters for neutrophil influx were identified for MWCNT (e.g., cluster 1: surface area, diameter, length and Co, and cluster 2: Mn and Mg), and multiple regression analysis indicated that for MWCNTs, neutrophil influx reduced as diameter increased [21].

In addition to these intratracheal instillation and pharyngeal aspiration studies, in-halation studies have also been undertaken comparing the effects of MWCNTs with different characteristics. For example, Gaté et al. [22] compared the effects of two inhaled MWCNTs, NM-401 (“long and thick”) and NM-403 (“short and thin”) in female Sprague–Dawley rats, using a nose-only exposure system with concentrations of 0.5 mg/m^3^ and 1.5 mg/m^3^ (2 × 3 h/day, 5 days/week for 4 weeks) and four post-exposure times (3, 30, 90, and 180 days) and found that both induced pulmonary neutrophil influx which was correlat-ed with deposited surface area dose (this was also seen if the materials were deposited in the lung via intratracheal instillation). To gain additional insights, a very thorough and detailed analysis of the whole lung transcriptome and the BALF proteome was undertaken and highlighted important differences between the effects of the two materials [23]. Inhalation of NM-401 and NM-403 altered the expression of genes involved in multiple signalling pathways, including immune system and inflammation pathways. They noted a good correlation between the number of differentially expressed genes (DEGs) and neutrophilic influx in all cases, which may be partly explained by the fact that many of the genes had a role in inflammation, but the high dose of NM-401 induced a higher number of DEGs than NM-403, which decreased over time, whereas the number of DEGs was comparable at all four post-exposure times for NM-403. The results indicated differential regulation of genes involved in fibrosis between the two nanomaterials, with greater effects for NM-401.

Overall, important progress has been made in linking some of the physicochemical characteristics of MWCNTs with specific biological endpoints. This has, unsurprisingly, given the potential for harm, been particularly well developed in relation to the possible mesothelioma hazard, with activities building upon the fibre paradigm associated with inhaled fibres, in particular asbestos, which identifies fibre biopersistence, length (typical-ly > 5 µm), and rigidity (typically related to diameter > 30 nm) as key factors in mesotheli-oma development following inhalation [24,25]. An Integrated Approach to Testing and Assessment (IATA) to support the grouping of High Aspect Ratio Nanomaterials (HARNs) based on their potential to cause mesothelioma has been developed [26], and this has been used in part to propose grouping of two MWCNTs (Mitsui-7 and NM-401) [27]. Questions still remain, however, on the general significance of a range of physico-chemical parameters of MWCNTs in relation to the range of possible ‘fibre’ and ‘particle’ related toxicological endpoints and, in particular, given the heterogenous nature of many of the aerosols produced using these materials, the importance of considering detailed pa-rameter distributions (e.g., distributions on aspect ratio) versus the use of average values [16].

With the primary objective of further exploring the effect of variations in physico-chemical properties on both acute and chronic pulmonary effects, using the more physiologically relevant inhalation exposure, in this study, two different MWCNTs were tested for pulmonary effects in vivo. Included was a ‘straight’ long (4 µm) and thick (70 nm) pristine MWCNT (pMWCNT) (other designations: NM-401, JRCNM04001a) and a chemically functionalized (COOH, 3.9 wt%), ‘tangled’, very long (10–30 µm) and thin (<8 nm) MWCNT (MWCNT-COOH) (other designation: JRCNM40004a).

Female Sprague–Dawley rats were whole-body exposed for 28 days to filtered air or MWCNT aerosols (pMWCNT (0.5 and 1.5 mg/m^3^) and MWCNT-COOH (1.5 and 4.5 mg/m^3^)). Animals were sacrificed at 3 times post-exposure: 3 days, 30 days, and 1 year. At all three time points, bronchoalveolar lavage fluid was analysed for inflammation (immune cell counts) and indicators of cytotoxicity and alveolar barrier damage (total protein, lactate dehydrogenase, and alkaline phosphatase). Histopathological analysis was undertaken using lung tissue sections semi-quantitatively at 3 days and 1 year post-exposure to 1.5 mg/m^3^ for both materials and qualitatively only for all other times and concentrations. Global mRNA expression levels in lung tissue (3 days post-exposure, 1.5 mg/m^3^) and concentrations of selected BALF cytokines and osteopontin were also assessed to identify any differences in responses at the molecular level. Standard optical microscopy and enhanced darkfield microscopy were used to explore the distribution and persistence of particles in the lung. Detailed characterisation of the delivered aerosols was undertaken to explore links between the physical characteristics of the aerosol particles and any biological effects.

Levels of deposition in the lung estimated from the measured aerosol characteristics using the MPPD model [28] were relatively low. BALF analysis indicated levels of pulmonary inflammation (neutrophil influx) broadly consistent with the deposited dose. Histo-pathological analysis indicated that both MWCNTs produced limited effects at the exposure levels used; however, global mRNA expression levels in lung tissue and BALF cytokines indicated different patterns for the two MWCNTs, potentially linked to the different characteristics of the aerosol particles for each material. A comparison of our results for pMWCNT with those from another very similar study using the same material (NM-401) but using nose-only exposure [22] indicates that small differences in the experimental system design can have a measurable effect on lung deposition and, thus, biological endpoints and that such factors are important to consider when assessing the results of such studies.

## 2. Materials and Methods

### 2.1. Multi-Walled Carbon Nanotubes

Carboxylated MWCNTs (JRC Reference JRCNM4004a), referred to here as MWCNT-COOH, were obtained from the Joint Research Centre (JRC, Ispra, Italy) and pristine MWCNTs (JRC Reference JRCNM04001a, OECD Reference NM-401), referred to here as pMWCNT, were obtained from the Fraunhofer Institute for Toxicology and Experimental Medicine (Hannover, Germany). MWCNT-COOH are characterised as very long (10–30 µm) and thin (<8 nm), and pMWCNT as long (4 µm) and thick (70 nm) (Table 1). The COOH content of MWCNT is 3.9% (Table 1). Metal contamination levels were measured using ICP-MS as described in Supplementary Information (Supplementary Information Table S1). MWCNT-COOH had a greater range and generally higher concentrations of impurities than pMWCNT, with Co and Fe the highest, 1720 and 632 ppm, respectively. The results for pMWCNT indicated all ≤10 ppm, except Fe at 3430 ppm.

### 2.2. In Vivo Exposure Study

The experiments were performed within the legal framework of the United Kingdom under a project licence granted by the Home Office of His Majesty’s Government. All procedures involving the animals were performed in accordance with the Animals (Scientific Procedures) Act 1986. Female pathogen-free Sprague–Dawley (SD) rats (9–13 weeks) were purchased from Harlan, UK. Rats were randomly assigned into groups (n = 6) and exposed to filtered air or aerosolized MWCNTs for 6 h per day for 5 days per week for 4 consecutive weeks. Naïve animal groups were also analysed (n = 4). For each type of MWCNT, two different aerosol concentrations were delivered. Target aerosol concentrations were 4.5 mg/m^3^, 1.5 mg/m^3^, and 0.5 mg/m^3^, referred to here as high, medium, and low, respectively. Medium and high concentrations were delivered for MWNCT-COOH. Unfortunately, difficulties aerosolising pMWCT prevented delivery of the high concentration, and thus, medium and low concentrations were delivered for pMWCNT. The concentrations used were chosen based on a consideration of the literature, as levels at which biological effects had been seen in other similar studies. This also usefully facilitated comparison with other relevant studies. During exposure, rats were housed individually in a 12 h/12 h light/dark cycle and had ad libitum access to food and water both during and post-exposure. Following exposure, the rats were returned to their cages. Biological effects were examined after 3-, 30-, and 365-days post-exposure (n = 4–6, depending on assay).

### 2.3. Whole-Body Exposure System

A schematic diagram and images of the overall setup for MWCNT aerosol generation and delivery to animals in a whole-body exposure system are shown in Supplementary Information (Figure S1). The aerosol generation system is described in more detail below. Both the aerosol generator and the exposure chamber (custom-made) were housed within separate compartments of a glovebox held at a slight vacuum (~0.7 kPa relative to the laboratory) to reduce contamination risk. Temperature and relative humidity were monitored within the glovebox. Animals were housed in individual cages within a custom-built exposure chamber of volume 0.375 m^3^.

Aerosol (or filtered air) flow into the exposure chamber was introduced through a baffle designed to distribute the air evenly throughout the chamber, ensuring each animal was exposed to the same aerosol concentration. This was verified prior to exposures using a test aerosol (NaCl, generated using a 6-jet Collison nebuliser), demonstrating that aerosol concentration varied by approximately ±4% and count median diameter by approximately ±2% at each of the animal breathing positions.

### 2.4. Aerosolisation of MWCNTs

MWCNT aerosols were produced using a NIOSH-developed acoustic aerosol generator [30]. Briefly, the system physically comprises a cylinder with latex diaphragms at the top and bottom forming a ‘drum’, into which MWCNT is placed, which during operation is agitated by a speaker located beneath the cylinder. Integral software controls the power and frequency of the amplification to the speaker and the bypass, generator, and exhaust flow rates (via mass flow controllers). The system employs active feedback to control the mass concentration (around a set target value) within the exposure chamber, which is monitored in real time close to the animal breathing zone using a DataRAM (pDR-1500, Thermo Fisher Scientific, Waltham, MA, USA), and also the exposure chamber and generator cylinder pressure, using a differential pressure transducer. Aerosolisation of MWCNT-COOH was straightforward; however, for pMWCNT, the action of the generator tended to produce very large agglomerates of the material that were not aerosolisable, and so reduced the quantity of material that could become airborne and, therefore, constrained the maximum deliverable concentrations. It is considered that this difference in behaviour of the two materials in the acoustic generator is due to their different physicochemical characteristics, but this was not investigated further. For filtered air control exposures, the generator was operated without CNT within the drum or operation of the speaker, but otherwise, the same control and feedback systems were used.

### 2.5. Characterisation of MWCNT Aerosols

The aerosol particle number size distribution and number concentration were continuously measured from a port on the exposure chamber using an aerodynamic particle sizer (APS Model 3321, TSI Inc., Shoreview, MN, USA) and condensation particle counter (CPC Model 3775, TSI Inc., Shoreview, MN, USA). Average aerosol mass concentration throughout each exposure was determined by sampling directly from the exposure chamber onto 37 mm filters mounted in air monitoring cassettes (Pall Corp., Port Washington, NY, USA), which were subsequently weighed using an ultra-micro balance (Model 238 SE6.6S-F with antistatic fan blower, Sartorius, Göttingen, Germany). Real-time aerosol mass concentration was monitored using the DataRAM, which also provided feedback to the acoustic generator software as described above. Aerosol mass size distributions were also measured at least once for each exposure group using a NanoMOUDI (Model 125-R, MSP, Shoreview, MN, USA).

The morphology of the aerosolised MWCNT particles was determined using high-resolution transmission electron microscopy (TEM) (JEOL 3000F, JEOL Inc., Tokyo, Japan). Whole-aerosol samples for TEM were taken directly onto 400 mesh copper TEM grids with lacey carbon film using a mini particle sampler (MPS, Ecomesure, Saclay, France) at a flow rate of 0.3 LPM for 3 min, sampling directly from the exposure chamber during one exposure for each MWCNT type. In addition, size-segregated TEM samples were obtained by loosely taping TEM grids to different stages of the NanoMOUDI for each MWCNT type.

### 2.6. Deposited Dose Estimation

Deposition fractions and deposited doses (in terms of both particle mass and surface area) in different parts of the respiratory tract were estimated using MPPD v3.04 (www.ara.com/mppd, accessed on 10 July 2024) [28]. The asymmetric Sprague–Dawley rat airway morphometry was used with default body weight (295 g), whole-body exposure condition, inhalability correction included, and clearance module not activated. Unless otherwise stated, default values determined by MPPD for the default body weight were used for physiological parameters (i.e., functional residual capacity (FRC) = 3.45 mL, upper respiratory tract (URT) volume = 0.41 mL, tidal volume = 2.08 mL, breathing frequency = 116 breaths/min, inspiratory fraction = 0.5).

**Figure 1:**
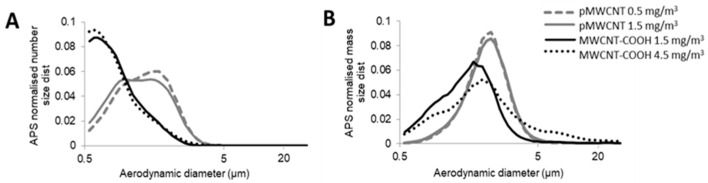
MWCNT aerosol characterisation: (**A**) particle number-based size distribution; (**B**) particle mass-based size distribution.

**Figure 2:**
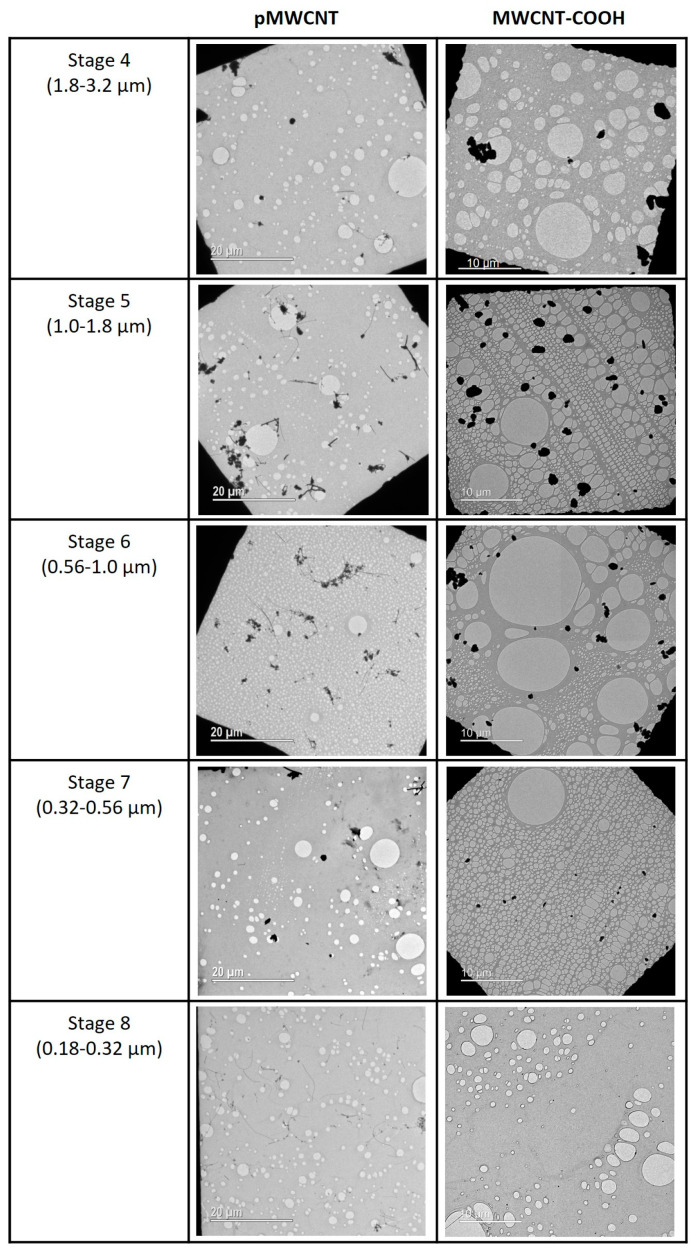
Representative TEM images of aerosol particles from key stages of NanoMOUDI impactor.

The Mass Median Aerodynamic Diameter (MMAD) was used in MPPD, as this is the most appropriate diameter metric for estimating mass deposited dose [4] in the particle size range where aerodynamic processes (sedimentation, impaction, interception) are likely to dominate [31]. Surface area deposition was obtained by scaling the estimated mass deposition by the specific surface area (Table 1). Aerosol parameters (MMAD, GSD) for input to MPPD were derived from APS measurements. Because the aerosol size distributions were clearly bimodal (Figure 1), we fitted two log-normal distributions to experimental data from each exposure (using QtiPlot, Iondev SRL, Romania, www.qtiplot.com, accessed 10 July 2024), for each of which MMAD and GSD and the relative mass in each were obtained (Supplementary Information Table S2) for use in estimating deposited doses using MPPD. We observed that MPPD reported slightly different results when using the Multimodal option as compared to two separate Unimodal runs combined, hence we chose to run MPPD with each individual aerosol mode separately, and summed the estimated deposited mass (or surface area) post-calculation. TEM images indicated MWCNT-COOH aerosols as agglomerates, broadly spherical in shape for all particle size ranges (Figure 2 and Supplementary Information Figure S2), with aspect ratios (AR) generally in the range 1 to 4. To determine deposition, an AR of 4 was used. The pMWCNT aerosol was much more complex, with single fibres of various diameters, complex fibre bundles, and broadly spherical particles (Figure 2 and Supplementary Information Figure S2). Aspect ratios ranged from 1 for spherical agglomerates to approximately 150 for the longer single fibres, with higher values for some of the thinner fibres. For the deposition calculations an aspect ratio of 30 was used, which was intended to broadly reflect the mixture with a greater weighting for the lower AR particles, which are considered more reflective of the (mass-weighted) average. This value is also consistent with that used in a previous study with the same material [22]. Aerosol effective density was estimated by iteration, i.e., changing the assumed density in the APS software (Aerosol Instrument Manager^®^, Version 8.1.0.0; TSI Inc., Shoreview, MN, USA) until the total aerosol mass concentration matched the observed gravimetric mass concentration. This approach resulted in estimated effective densities of 0.032 g/m^3^ for pMWCNT and 0.071 g/m^3^ for MWCNT-COOH.

As indicated above, estimating deposition using MPPD requires assumptions to be made for a large number of parameter values, including those relating to the aerosol and the animal model. Many of these have a significant degree of associated uncertainty, especially for complex aerosols [31]. To explore the effect on deposited dose estimates of variations in a number of key parameters (e.g., breathing rates, aerosol particle effective density, and aspect ratio), a limited sensitivity analysis was undertaken (see Appendix A for details) to support the interpretation of the results.

### 2.7. Bronchoalveolar Lavage and Lung Tissue Collection

Rats were sacrificed by exsanguination by cardiac puncture under isoflurane anaesthesia (induced at 5%, maintained at 1.5–2% in 100% oxygen). Bronchoalveolar lavage fluid (BALF) was collected via tracheal cannula with 2 × 7 mL aliquots of PBS. BALF was centrifuged at 1500× *g* for 10 min. Cells from both aliquots were pooled for analysis of total and differential cells. The BALF supernatant from the first wash was retained for gross toxicity analysis.

Following BAL, the apical and azygous lung lobes were tied off and snap frozen in liquid nitrogen for transcriptomic analysis. The remainder of the lungs were removed and inflated and fixed with freshly made 4% paraformaldehyde via tracheal cannula, at a pressure of 30 cm of water, and then processed into paraffin-embedded tissue blocks. Serial lung sections 5 μm thick were cut onto microscope slides for further processing and analysis.

### 2.8. Bronchoalveolar Lavage Analysis

Cells from both BALF aliquots were pooled for total and differential cell analysis. The cells infiltrated in the BALF were resuspended in PBS, and the total number was determined with a Neubauer hemacytometer under a microscope. BALF cells were then centrifuged for 5 min at 500 rpm and pelleted onto slides using a cytocentrifuge. Slides were air-dried at room temperature and stained using the Shandon Kwik-Diff Stains kit (Thermo Scientific, Waltham, MA, USA), and cell differentials were counted by microscopic observation by individuals blinded to treatment. At least 500 cells on the slides were counted and identified as macrophages, neutrophils, eosinophils, basophils, and lymphocytes based on morphological criteria. Samples were classified as outliers based on total cell counts and neutrophil percentages using the Robust Outlier Test (ROUT) method with a false discovery rate of 0.05. These outlier samples were excluded from the BAL analysis and other biological endpoint assessments.

Total protein, lactate dehydrogenase (LDH), and alkaline phosphatase (ALP) (indicators of cytotoxicity and alveolar barrier damage) were determined using the supernatant of the first lavage wash. Total protein was measured using Bio-Rad protein assay kit (#5000001), and ALP was measured using Abcam alkaline phosphatase assay kit (#ab83369). For the LDH assay (Promega, Madison, WI, USA), 50 µL of BALF was added to 50 µL of reconstituted substrate solution and incubated for 30 min at room temperature in the dark. Then, 50 µL of stop solution was added to each well, and the absorbance was measured at 492 nm (Ab_492_). Relative LDH in BALF was determined using the following equation.

$$Relative\;LDH\;in\;BALF=\frac{Ab_{492}\left(Exposed\right)-Ab_{492}\left(PBS\right)}{Ab_{492}\left(Unexposed\right)-Ab_{492}\left(PBS\right)}$$

To explore particle clearance, brightfield microscope images of BALF macrophages for groups exposed to 1.5 mg/m^3^ of each type of MWCNT were examined for the presence of MWCNTs at each post-exposure time point, and the percentage of ‘pigmented’ cells was assessed (n = 4–6).

### 2.9. RNA Extraction from Lung Tissues

Lung tissues were homogenised in 8 mL/g (v/w wet mass) methanol and 2.5 mL/g (v/w) water using a bead-based homogeniser (Precellys 24; Stretton Scientific, Stretton, UK). Lung homogenate aliquots were then taken for RNA extraction using Qiagen’s mini RNeasy Kit and QIAshredder (Qiagen, Crawley, UK) according to the manufacturer’s protocol. RNA was quantified with a NanoDrop 1000 spectrophotometer (Thermo Scientific, Waltham, MA, USA).

### 2.10. Transcriptomic Analysis

RNA quality was determined using an Agilent 2100 Bioanalyser, and those samples with RIN above 8.0 were used for library preparation. For mRNA sequencing analysis, sample processing and sequencing were carried out in conjunction with Earlham Institute (UK) using Illumina HiSeq4000 150PE. A total of 20 HT Stranded RNA libraries were constructed and sequenced on 1 lane of the HiSeq_4000 with a 150PE read metric. HiSeq4000—250–300 million reads per lane for each direction were sequenced. Raw data as FASTQ files (one file per read direction for each barcoded library) were obtained. Raw sequence data were then imported, and paired data were processed for RNA-Seq analysis using CLC Genomics Workbench 20.0 (https://digitalinsights.qiagen.com, accessed on 27 July 2020) by annotation using the Rnor_5.0 (Ensembl release 79) rat reference genome build. All annotated transcripts were extracted (using an mRNA track), then the reads were mapped against all the transcripts and to the whole genome using the default setting. From this mapping, the reads were categorized and assigned to the transcripts using the EM estimation algorithm, and expression values for each gene were obtained by summing the transcript counts belonging to the gene. Further comparative analysis and visualisation of differentially regulated transcripts were carried out using Qlucore Omics Explorer 3.8 (Qlucore, Lund, Sweden). Principal component analysis (PCA) enabled visualisation of any similarities and/or differences in gene expression between sample groups. Samples identified as outliers based on the expression of highly regulated genes that are not typically expressed in lung tissues were excluded from further analysis. Differentially expressed gene lists (*q*-value < 0.05 and log2 fold change > 1.0) were derived.

### 2.11. Selected Cytokine Expression

BALF was assessed for protein levels of selected inflammatory cytokines, including IL-1β (Interleukin-1β), Cxcl1 (CINC-1, cytokine-induced neutrophil chemoattractant 1), and Ccl2 (MCP-1, monocyte chemoattractant protein-1) using DuoSet kits, and Osteopontin (OPN or SPP1, secreted phosphoprotein 1), as a marker of potential pro-fibrotic changes, using Quantikine ELISA kit from R&D systems (Abingdon, UK) according to the manufacturer’s specifications. These were chosen as previous studies have indicated their relevance and sensitivity to the assessment of particle effects in the lung, e.g., [32]. Samples were assessed following the instructions and analysed in duplicate. Absorbance was assessed at 450 nm with background levels at 570 nm using a Bio-Tek Synergy HT plate reader (BioTek Instruments, Inc., Winooski, VT, USA).

The mRNA levels of selected cytokines, including *IL-1β*, *Cxcl1*, *MCP-1*, and *Spp1* in lung tissue were determined based on the transcriptomic analysis. The expression levels were represented by the RPKM values (Reads Per Kilobase of transcript per Million mapped reads) for each specific gene. Expression values were then normalized to those of the air-exposed group.

### 2.12. Immunofluorescence on Lung Tissue Sections

Paraffin-fixed lung tissue sections were processed by deparaffinization and dehydration. Antigen retrieval was performed by immersing slides in 10 mM citric acid (pH adjusted to 6.0) and microwaving at high power (~850 W) for 20 min. Then slides were blocked using 1% PBS/BSA (Sigma Aldrich, St. Louis, MO, USA) for 30 min. Then, slides were incubated with primary antibody anti-Osteopontin antibody (goat anti-mouse, Abcam, ab11503, 1:100 constituted in 1% PBS/BSA) for 60 min before incubation with secondary antibody Alexa Fluor 488 (1:200 diluted in 1% PBS/BSA) for 60 min. The slides were then dried following PBS wash before mounting using 50 μL Vectashield. Nail varnish was used to seal the coverslip. Slides were observed using the Zeiss LSM9 confocal microscope system with ZEN software within 72 h to obtain optimal results.

### 2.13. Histopathology

Lung tissue sections were processed for histopathological analysis. Two staining protocols were used: the Hematoxylin & Eosin (H&E) procedure for general histopathology and the Trichrome-Masson method for the deposition of collagen (a marker of fibrosis). A qualitative assessment of H&E-stained lung tissues from all exposed groups at all time points was undertaken. In addition, animals exposed via inhalation to 1.5 mg/m^3^ of both types of MWCNT, along with their matched controls, were analysed for histopathological severity scoring. The analysis was performed by researchers blinded to the treatment groups, using brightfield microscopy to examine tissue sections from both the 3-day and 1-year post-exposure groups. A semi-quantitative approach was taken to evaluate the changes in the alveoli (oedema, proliferation of macrophages, fibrosis, and presence of pigment in the alveolar macrophages), bronchioles (club cell proliferation and loss of cilia) and interstitium (oedema and fibrosis), based on a scoring system as follows: 0 = none, 1 = minimal, 2 = mild, 3 = moderate, 4 = marked/severe.

### 2.14. Enhanced Dark-Field Microscopy

MWCNTs were detected in H&E-stained lung tissue sections using the Cytoviva enhanced darkfield hyperspectral system with an Olympus BX 43 microscope and a Qimaging Retiga4000R camera (Cytoviva, Auburn, AL, USA). A section of the right caudal lung lobe was partially scanned at 40× enhanced darkfield mode by a manual scan of the longest tissue axis and a scan perpendicular to the longest axis. The right caudal lung lobes were analysed 3 days and 12 months post-exposure (n = 3–6 samples per exposure group and n = 2 samples per control group). Enhanced darkfield images were acquired at 40× and 100×. Image intensity levels were adjusted in Adobe Photoshop to allow simultaneous visualisation of low intensity tissue and high intensity MWCNTs.

### 2.15. Statistical Analysis

All data are presented as mean ± standard deviation for 4–6 animals unless indicated otherwise. Statistical significance was evaluated using a two-tailed Student’s *t*-test unless otherwise stated. Where indicated, one-way ANOVA and Dunnett’s post-test analysis were undertaken using GraphPad Prism 9.

## 3. Results

### 3.1. Aerosol Characterisation and Deposited Doses

A summary of the aerosol characteristics is presented in Table 2, and the aerosol particle size distributions are presented in Figure 1. The MMAD for both concentrations of MWCNT-COOH was 2.2 µm, very similar to that for the 1.5 mg/m^3^ pMWCNT, at 2.0 µm, although the value for the 0.5 mg/m^3^ aerosol was somewhat smaller at 1.6 µm. All indicate a respirable aerosol. These results were derived from the APS data. Some limited NanoMOUDI measurements were also undertaken, and these indicated somewhat lower MMAD values for both material aerosols, but with significantly wider distributions, i.e., pMWCNT, 1.0 µm (GSD 4.5), and MWCNT-COOH, 1.1 µm (GSD 5.2). These differences arise, in part, from differences in the measurement techniques. The APS values have been used for the deposition modelling as continuous measurements were taken for all exposures, whereas NanoMOUDI measurements were only undertaken a few times for each material and are, therefore, considered potentially less representative. Electron microscopy images of the aerosol particles (Figure 2 and Supplementary Information Figure S2) indicate that the MWCNT-COOH exist predominantly as approximately spherical particles of tangled fibres, occasionally with a protruding tube, whilst the pMWCNT particles exist both as single fibres and as agglomerates of a range of shapes.

The NanoMOUDI impactor allowed collection of aerosol particles on TEM grids for visualisation on 13 stages (aerodynamic size cut-offs (nm): 10,000, 5600, 3200, 1800, 1000, 560, 320, 180, 100, 56, 32, 18, 10). As expected from the particle size distribution, the majority of particles were found on Stages 4 to 8 (range 1.8 µm–180 nm). TEM images from the different stages show the morphology and structure of the aerosolised MWCNT changes with aerodynamic particle size. MWCNT-COOH aerosols are broadly spherical in shape, albeit often elongated (with aspect ratios ranging from approximately 1 to 4), with no evidence of individual fibres observed. There is some evidence that the larger aerosols (Stages 4–6, aerodynamic diameter 0.56–3.2 µm) may be comprised of aggregates of smaller bundles of similar shape. As aerodynamic size decreases, the number of MWCNT-COOH particles decreases, broadly in line with the observed aerosol size distribution, with very few particles observed on Stage 8 and below. The pMWCNT aerosol is a much more complex mixture of both broadly spherical and elongated fibres. Some particles clearly contain multiple fibres, many have bundled groups of CNT attached to fibres, and the fibres themselves have a range of thicknesses (the majority in the range 50–100 nm, broadly in line with manufacturer information [29], but diameters as small as 7 nm and as large as 700 nm were observed across TEM images for pMWCNT). The thicker fibrous aerosols, with and without attached bundles, appear mostly on Stages 5 and 6 (0.56–1.8 µm), broadly spherical aerosols without attached fibres on Stages 6 and 7 (0.32–1.0 µm), whereas thinner, individual fibres, many >10 µm, without attached bundles are more prominent on Stage 8 (0.18–0.32 µm). These TEM images highlight the inherent complexity in characterising (some types of) MWCNT aerosol and in determining which properties are linked with any toxicological adverse outcomes observed.

Estimates of the deposition fractions in different parts of the respiratory tract and total deposited doses (mass and surface area) generated using the MPPD model are provided in Table 3. The modelling estimates indicated low levels of deposition overall, but that the MWCNT-COOH aerosol was significantly more effectively deposited than the pMWCNT aerosol. The results indicate that the majority of the deposition occurs within the head airways for both materials (deposition fractions 0.27–0.39). We estimated that 54 and 109 µg of pMWCNT were deposited within the thoracic region during one month of inhalation of 0.5 and 1.5 mg/m^3^, respectively. For MWCNT-COOH, the estimated thoracic deposited doses were 102 and 288 µg during one month of inhalation of, respectively, 1.5 and 4.5 mg/m^3^. The thoracic deposited doses for the two materials at 1.5 mg/m^3^ were very similar. The ratio of the deposited doses for the two concentrations of MWCNT-COOH reflected the ratio of the concentrations (i.e., 3), whereas for pMWCNT, the higher concentration only resulted in a doubling of the deposition, reflecting differences in the aerosol characteristics at the two concentrations. This pattern was repeated for the pulmonary mass doses. The pulmonary deposition fractions were low but differed significantly for the two materials, i.e., 1.4–1.6% and 0.01–0.04% for MWCNT-COOH and pMWCNT, respectively. Consequently, the pulmonary mass doses were markedly lower for pMWCNT, at only 15% of the MWCNT-COOH dose for the 1.5 mg/m^3^. Surface area deposited doses in the pulmonary region were determined by multiplying the mass doses by the specific surface area of the material (Table 1), and again revealed marked differences between the two materials, with, at 1.5 mg/m^3^, the deposition of pMWCNT only 0.5% of the deposition of MWCNT-COOH. The estimates were determined using MPPD, assuming the default rat weight of 295 g, broadly consistent with the experimental animal weights. Approximate values of deposition doses in terms of rat body weight (mg/kg bw) can be estimated using this value (e.g., 0.02 mg/kg bw and 0.14 mg/kg bw, respectively, for pMWCNT and MWCNT-COOH at 1.5 mg/m^3^). Similarly, approximate deposited doses per unit lung mass can be estimated using a lung weight of 1.5 g [33].

Determining deposition using MPPD requires assumptions to be made about a large number of parameter values, including those relating to the aerosol and the animal model. Many of these have a significant degree of associated uncertainty. To explore the effect on deposited doses of variations in a number of key parameters (breathing rates and aerosol particle effective density and aspect ratio), a limited sensitivity analysis was undertaken (see Appendix A for details). Plausible differences in breathing rates had a minimal effect on deposited dose estimates for either material (ratio of variant condition to base case ranged from 0.6 to 1.3, Table A3). For MWCNT-COOH, the ranges in particle density and aspect ratio also had a limited impact (ratio of variant condition to base case ranged from 0.4 to 1.7, i.e., less than a factor of 2, Table A1 and Table A2). The effects of the considered ranges on effective density and aspect ratio on deposition for pMWCNT were much greater (ratio of variant condition to base case 1.0 to 7.6 for effective density and 0.1 to 10 for aspect ratio, Table A1 and Table A2), reflecting the more complex aerosol.

### 3.2. Bronchoalveolar Lavage Fluid Cytology and Biochemistry

**Figure 3:**
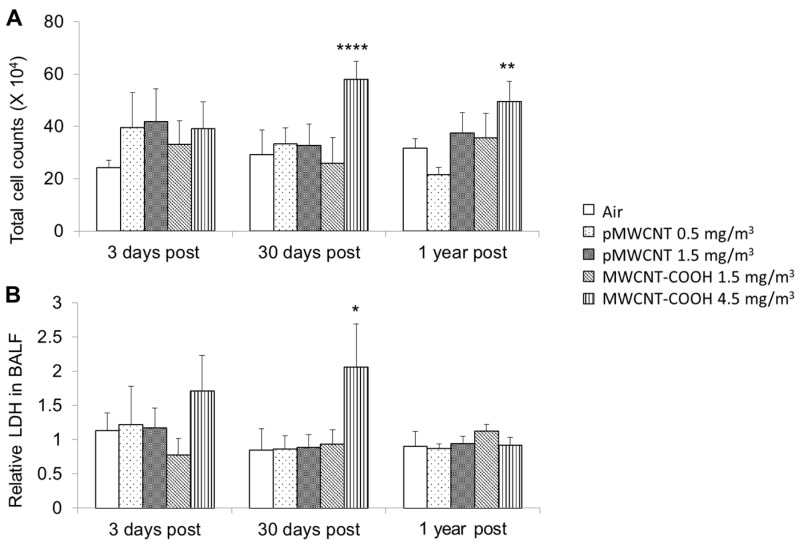
Toxicity analysis: (**A**) total cell counts and (**B**) LDH levels in bronchoalveolar lavage fluid (BALF). Results for LDH are normalised to their distinct unexposed control groups. Significance was determined by one-way ANOVA versus air-exposed groups with Dunnett’s post-test: *, *p* < 0.05, **, *p* < 0.01, and ****, *p* < 0.0001.

Results from the counting of cells recovered from bronchoalveolar lavage (BAL) fluid indicate no change in total cell numbers for any of the experimental groups up to 12 months post-exposure, except for MWCNT-COOH at high exposure concentration (4.5 mg/m^3^), which showed a significant increase that peaked at 30-days post-exposure and recovered somewhat by 1 year (Figure 3). No significant changes in percentages of macrophages were seen for any groups (Supplementary Information Figure S3). The % neutrophils was low for all groups, i.e., no group (exposed or control) had neutrophils > 2.5% of the total cell numbers, and no exposed groups had % neutrophils significantly greater than controls. Neutrophil counts (derived by multiplying total cell counts by % neutrophils) were only significantly greater than air control for MWCNT-COOH at 4.5 mg/m^3^ (*p* < 0.05), which is consistent with the difference in total cell numbers. Concentrations of LDH (assay of cellular integrity) were higher only for the high concentration (4.5 mg/m^3^) for MWCNT-COOH at 30 days post-exposure, with resolution by 1 year. No changes were seen in total protein and ALP in BALF for either material at any concentration (Supplementary Information Figure S4). The results for MWCNT-COOH (e.g., total cell counts and LDH at 30 days greater at 4.5 mg/m^3^ than 1.5 mg/m^3^) are suggestive of a dose-dependent effect, but further experiments with a wider range of concentrations would be required to confirm this.

### 3.3. Histopathological Analysis

**Figure 4:**
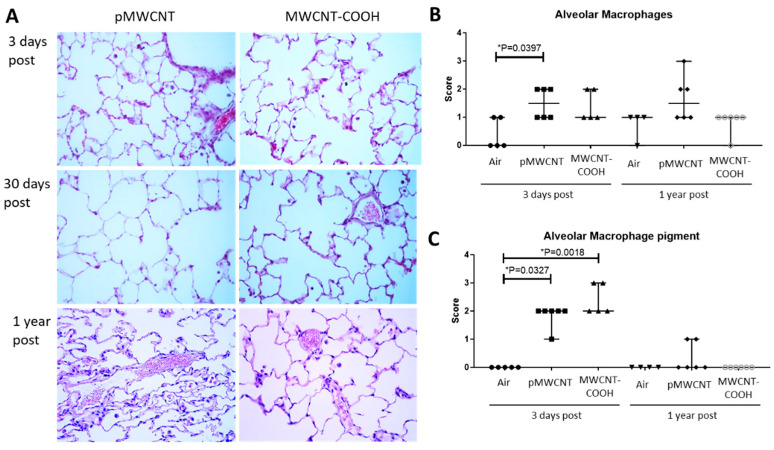
Histopathology analysis for rats exposed to the medium aerosol concentration (1.5 mg/m^3^) for both MWCNTs: (**A**) representative H&E-stained lung sections; (**B**) semi-quantitative assessment of alveolar macrophage numbers; and (**C**) semi-quantitative assessment of levels of pigmented macrophages. A *p*-value of less than 0.05 was considered statistically significant and is indicated by an asterisk (*).

Histopathological analysis was performed based on observation of lung tissue sections with Hematoxylin & Eosin (H&E) staining for general histopathology and Trichrome-Masson staining for collagen (a marker of fibrosis). A qualitative assessment of H&E-stained lung tissue sections for all concentrations and time points identified no significant histopathology (e.g., Figure 4A for 1.5 mg/m^3^). A systematic semi-quantitative analysis of H&E-stained lung tissues from 3 days and 1 year post-exposure for the medium aerosol concentration (1.5 mg/m^3^) for both MWCNTs indicated no significant histopathological changes (Table 4). However, the number of alveolar macrophages seems to be slightly increased for both types of MWCNTs at 3 days post-exposure and pMWCNT at 1 year post-exposure in comparison to controls, although this difference was only significant for pMWCNT at 3 days (Figure 4B). A similar pattern of changes was observed in alveolar macrophages pigmented as a result of internalised or associated MWCNTs (Figure 4C). Pigmented macrophages were seen for both materials at 3 days, but none (MWCNT-COOH) or much reduced (pMWCNT) at 1 year, indicating clearance from the lung (Figure 4C).

There was no significant change in the deposition of collagen as evidenced by Trichrome-Masson staining (Supplementary Information Figure S5), supporting the lack of a fibrotic effect at the tested exposures.

### 3.4. Pulmonary Distribution of Deposited MWCNTs and Biopersistence

**Figure 5:**
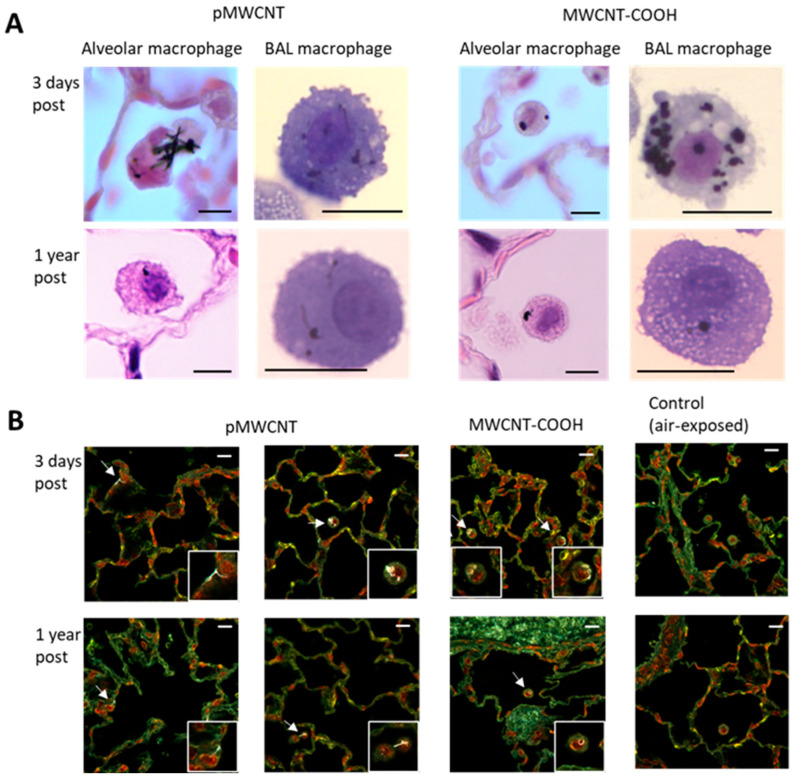
Localisation of inhaled particles in pulmonary tissues of rats exposed to the medium aerosol concentration (1.5 mg/m^3^) for both MWCNTs at 3 days and 1 year post-exposure: (**A**) brightfield microscopy and (**B**) enhanced darkfield microscopy. The inset images highlight areas containing MWCNTs, as indicated by the arrows. Scale bars, 10 µm (**A**) and 20 µm (**B**).

Many of the MWCNTs deposited in the lungs could be observed directly by brightfield microscopy, as single- or multi-fibre agglomerates internalised within or associated with cells, in particular alveolar macrophages and macrophages recovered from BALF (Figure 5A). The morphology of observed deposited pMWCNT particles is complex and includes single fibres, fibre agglomerates, and small spheres with and without protruding fibres, whereas for MWCNT-COOH, the majority of particles are broadly spherical. This is consistent with the range of particle shapes seen in the delivered aerosol for each material (Figure 2).

A semi-quantitative assessment of the fraction of BALF macrophages containing MWCNT particles following exposure at the medium concentration (1.5 mg/m^3^) was undertaken for each time point, which indicated approximately 80% for both MWCNTs at 3 days, reducing to 40–60% at 30 days and very few at 1 year post-exposure for both materials (Supplementary Information Figure S6). This indicates significant particle clearance during the post-exposure period. This is consistent with findings from the histopathology sections, which indicate significant numbers of macrophages containing particles (pigmented) at 3 days, reducing close to control levels by 1 year (Figure 4C). However, these standard microscopy approaches may underestimate the total retained dose as only easily visualised particles are identified, and sometimes there are multiple small particles in a single macrophage.

To visualise the deposited MWCNT particles in different pulmonary regions (including airways, alveolar macrophages, terminal bronchioles, interstitium, and lymphocytic tissue), enhanced darkfield microscopy was used. This technique allows improved detection of individual MWCNTs in tissue sections (Figure 5B). It should be noted that this imaging was undertaken using tissue sections from lavaged lungs, so some immune cells will have been extracted; however, using the BAL procedure adopted here would typically have resulted in the removal of <70% of the total immune cells [34], so many immune cells were still clearly visible within the sections and it remained possible to characterise their localisation within the lung in a qualitative manner. The BAL procedure would also have no impact on the presence of particles within the interstitium. The enhanced darkfield microscopy images revealed that, in addition to mainly being phagocytized by alveolar macrophages, single or agglomerates of MWCNTs were also present in the interstitium at the time points analyzed, including both 3 days and 1 year post-exposure (Supplementary Information Table S3). At 3 days post-exposure, both types of MWCNTs appeared evenly distributed over the lung lobe section, with no indication of increased deposition or accumulation at terminal bronchioles or in perivascular/peribronchiolar (lymphatic draining) regions. pMWCNTs, appearing as fibres with some bent, single, and bundled, thin and thick fibres, occasionally associated with compact non-fibre agglomerates, were mainly observed in macrophages, but also at or in alveolar walls (interstitial), whereas MWCNT-COOH, appearing as compact micro-granular agglomerates, were almost exclusively observed in macrophages, but detection in interstitium is difficult due to a higher background scatter (i.e., the agglomerates of MWCNT-COOH absorb light in the centre (black) and scatter light at the periphery (white) in enhanced darkfield, similar in appearance to common artefacts in tissue samples). At 1 year post-exposure, many fewer MWCNT-COOH agglomerates could be observed in the partial scan, compared to pMWCNT. There was no sign of pulmonary accumulation for either type of MWCNT, besides the retention of a small fraction of pMWCNT in the interstitium and in macrophages, and, occasionally, close to blood vessels (perivascular). Individual macrophages typically only contained a few MWCNTs and did not appear overloaded or immobilised in clusters, indicating that they were still involved in alveolar clearance 12 months post-exposure.

### 3.5. Transcriptomic Profiles of the Lung, Selected Cytokines, and Immunofluorescent Staining for Osteopontin

**Figure 6:**
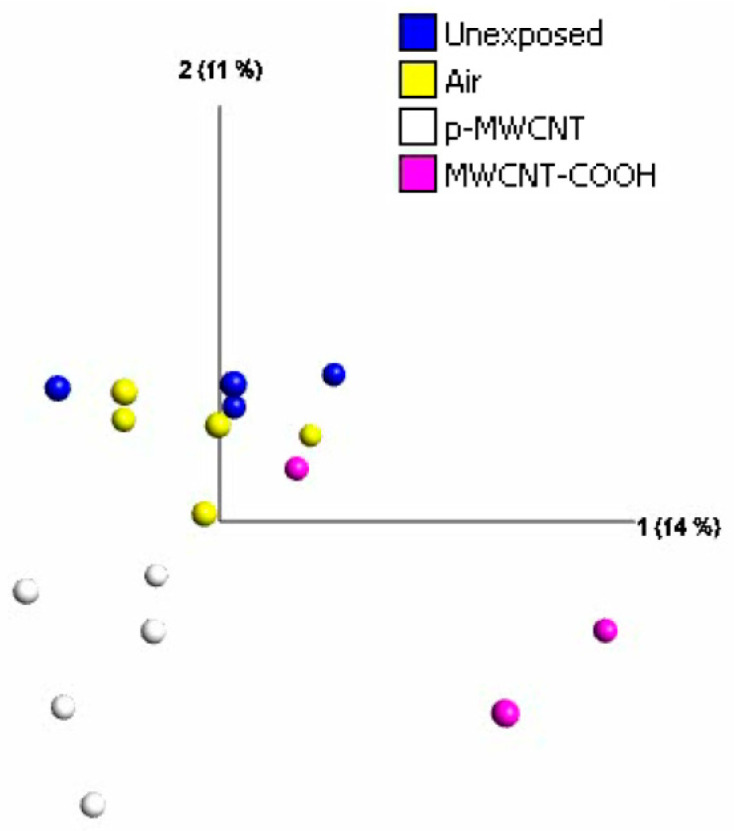
Principal component analysis (PCA) score plots of lung tissue gene expression from rats exposed to air and two MWCNT aerosols for the medium aerosol concentration (1.5 mg/m^3^) for both MWCNTs at 3 days post-exposure (unexposed group n = 4, exposed groups n = 6, with outliers (see Section 2.10) excluded).

As shown above, no histopathological changes were seen, and pulmonary inflammation was only observed at the highest deposited dose (i.e., MWCNT-COOH at 4.5 mg/m^3^). Therefore, more sensitive and high-throughput methods, including transcriptomics, were applied to help understand the molecular mechanisms triggered after exposure to such nanomaterials in relation to their physico/chemical properties. The principal component analysis (PCA) score plot from the transcriptomic analysis of 3-day post-exposure groups (1.5 mg/m^3^) demonstrates a clear separation of pMWCNT exposure groups from all other groups, confirming that pMWCNT induced perturbations to gene transcription more than MWCNT-COOH (Figure 6). Further statistical analysis was applied to the gene expression data to investigate the effects induced by exposure to both types of MWCNTs at the same aerosol concentration (air exposure group as the control). Following MWCNT-COOH exposure, only 14 genes were found to be differentially expressed, whereas for pMWCNT, 34 differentially expressed genes were identified (*q* < 0.05 and log2-fold change > 1.0) (Supplementary Figure S7). However, there were no overlapping genes regulated in both groups, indicating different perturbation levels to gene transcription induced by these two types of MWCNTs at the examined exposure dose or indicative of a high signal-to-noise ratio due to low expression levels. Reducing the fold-change cut-off to 1.5 increased the number of differentially expressed genes to 49 and 76 for MWCNT-COOH and pMWCNT, respectively, but again, there were no overlapping genes. The differentially expressed genes induced by pMWCNT exposure (listed in Supplementary Figure S7) can be broadly characterised as being involved in pro-inflammatory processes. The gene expression pattern following exposure to MWCNT-COOH was much less clear (Supplementary Information Figure S7), at a level typical of noise.

**Figure 7:**
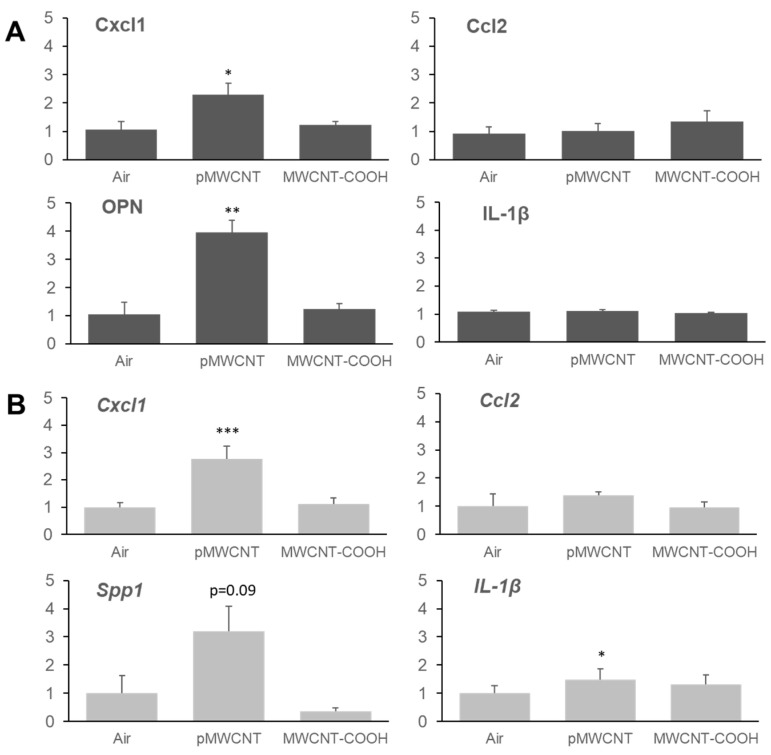
Expression of cytokines IL-1β, Cxcl1, Ccl2 (MCP-1), and OPN from rats at 3 days post-exposure to the medium aerosol concentration (1.5 mg/m^3^) of two types of MWCNT aerosols (normalized to air control): (**A**) protein levels in BALF and (**B**) mRNA levels in lung tissue. Significance is tested in relation to air-exposed groups, and a *p*-value of less than 0.05 was considered statistically significant: *, *p* < 0.05; **, *p* < 0.01; and ***, *p* < 0.001.

Effects on several cytokines and chemokines relevant to pro-inflammatory and fibrotic responses, chosen for their relevance and sensitivity to the assessment of particle effects in the lung [32], were assessed by measuring their protein concentrations in BALF (Figure 7A) and mRNA concentrations in the lung tissues (Figure 7B) at 3 days post-exposure to 1.5 mg/m^3^. For MWCNT-COOH, there were no significant changes in any levels in comparison to air control. For pMWCNT, there was no significant change in IL-1β or MCP-1(Ccl2) concentrations, whereas there were significant increases in Cxcl1 and osteopontin (OPN, secreted phosphoprotein 1 or SPP1) in comparison to air control. Analysis at other time points for pMWCNT indicated OPN levels remained above control levels at 30 days post-exposure, resolving by 1 year, and Cxcl1 levels resolved to control levels by 30 days (Supplementary Information Figure S8).

**Figure 8:**
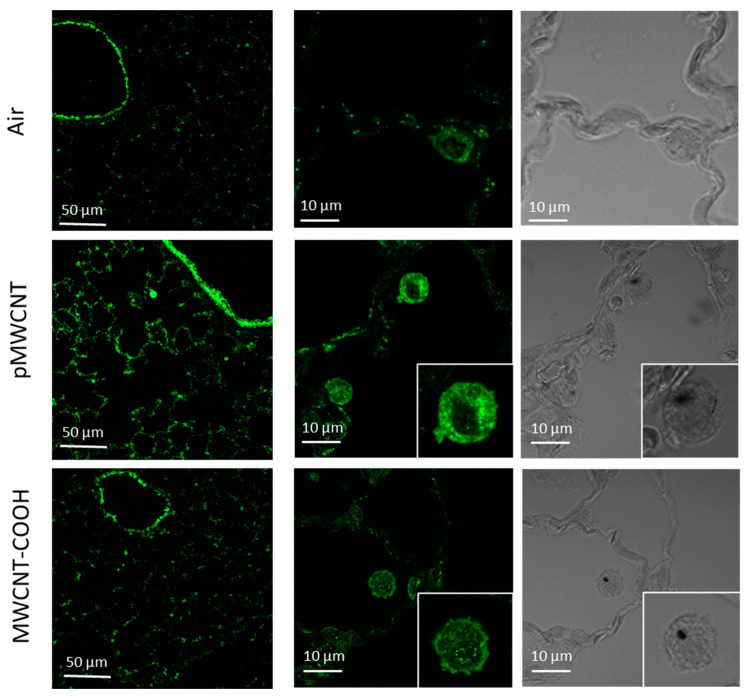
Immunofluorescence staining of OPN in lung tissues from rats at 3 days post-exposure to air (control) and the two types of MWCNT aerosols at the medium aerosol concentration (1.5 mg/m^3^). Fluorescence images were captured using consistent parameters.

Osteopontin (OPN), a glycoprotein secreted by various cell types, including inflammatory, immune, fibroblast, osteoblast, and cancer cells, is believed to play a key role in CNT-induced lung fibrosis [35] and emphysema [36]. The OPN expression in lung tissues was further explored by applying immunofluorescence to identify the effects of inhaled MWCNTs at 3 days post-exposure (Figure 8). Increased expression of OPN in lung tissues, particularly in the alveolar macrophages, in comparison to air exposure, was found after exposure to pMWCNT, but not MWCNT-COOH, consistent with the cytokine and mRNA results and indicating potential pre-fibrotic effects induced by the pMWCNT.

## 4. Discussion

### 4.1. Pulmonary Toxicity of MWCNT-COOH and pMWCNT Aerosols

Whole-body exposure of female Sprague–Dawley rats for 6 h/d for 28 days to acoustically-generated aerosols of two MWCNTs (pMWCNT at 0.5 and 1.5 mg/m^3^ and MWCNT-COOH at 1.5 and 4.5 mg/m^3^) produced no significant histopathological changes at any of the 3 post-exposure times (3 d, 30 d, and 1 year) (Table 4, Figure 4). Exposure to pMWCNT at both 0.5 and 1.5 mg/m^3^ also had no significant effect, in comparison to controls, on BALF cell counts and LDH, TP, and ALP levels at any post-exposure time. Similarly, exposure to MWCNT-COOH at 1.5 mg/m^3^ also had no impact on markers of effect in BALF. However, MWCNT-COOH at the higher concentration of 4.5 mg/m^3^ induced total BALF cell counts above control levels at 30 days and 1 year (Figure 3) and LDH levels in BALF above controls at 30 days (Figure 3), suggesting increased membrane permeability up to 30 days post-exposure. These data could be simplistically interpreted as suggesting a NOAEL of 0.5 mg/m^3^ for the pMWCNT aerosol and 1.5 mg/m^3^ for the MWCNT-COOH aerosol.

These results are consistent with those from other 28-day inhalation studies using different MWCNT materials which have, for example, identified no effects on BALF immune cell counts at 0.4 mg/m^3^ of one MWCNT aerosol (well dispersed nebulizer gener-ated aerosol with >70% individual fibres, geometric mean diameter 63 nm and length 1.1 µm) [37] and 0.2, 0.5, and 1.0 mg/m^3^ of another MWCNT aerosol (atomization generated aerosol with aerosolised fibres ranging in length from 68–1517 nm with a count median length of 330 nm diameter 10–15 nm) [38]. However, other 28-day studies have seen adverse effects at similar or lower concentrations than used here. Kim et al. [39] undertook a 28-day inhalation study with ‘tangled’ MWCNTs aerosolised using an acoustic generator. The characterisation indicated individual fibers with diameters ranging from 5–10 nm and aerosol agglomerates (MMAD 381–1015 nm). Aerosol concentrations of 0.26, 1.44, and 4.25 mg/m^3^ were used, and significant changes in neutrophils and LDH levels and granulomatous lesions were seen at the medium and high concentrations, all suggesting a NOAEL of 0.26 mg/m^3^. This variation in biological effects for different MWCNT aerosols primarily reflects differences in the physical characteristics of the aerosol particles, which determine the levels of deposition, and differences in physicochemical characteristics, which influence the biological effects of the deposited particles. However, other experimental factors may also influence the results; for example, Kim et al. [39] used male rats, and there has been some indication of a greater sensitivity of male vs. female rats [40]. Thirteen-week inhalation exposure studies with various MWCNTs have also seen significant biological effects at lower concentrations than those used here (i.e., NOAELs from <0.1–0.2 mg/m^3^) [40,41,42], which is unsurprising, as biological effects are linked to accumulated deposited doses in the lung and longer duration studies will result in higher deposited doses.

### 4.2. Comparison of the Toxicity of Lung Deposited MWCNT-COOH and pMWCNT

As indicated previously, the physical characteristics of MWCNT aerosol particles determine the level of deposition in the respiratory tract, and the physicochemical characteristics of the deposited particles drive the biological effects (per unit deposition). To allow comparison across inhalation studies, where differences in aerosol characteristics may lead to differences in levels of deposition, it is recognised that information on lung deposition is required to interpret study results and, as such, this is a general requirement of OECD Inhalation Test Guidelines [43,44]. Ideally, measurements of lung burdens would have been undertaken here, but this is challenging for carbon nanomaterials [45,46], so in this study, we have relied on modelling results to estimate total levels of deposition. The modelling estimates indicated low levels of deposition overall, but the MWCNT-COOH aerosol was significantly more effectively deposited than the pMWCNT aerosol (Table 3), with pulmonary deposition fractions of, respectively, 1.4–1.6% and 0.01–0.04%. The estimated pulmonary deposited mass dose at 1.5 mg/m^3^ was 7 times higher for MWCNT-COOH than pMWCNT (41.5 µg vs. 6.1 µg), and, due to the greater specific area, the surface area dose was 200 times higher (208 cm^2^ vs. 1.1 cm^2^). Unfortunately, due to the lack of histopathological changes seen for any concentration, and changes to BALF cell counts and LDH levels only seen for MWCNT-COOH at 4.5 mg/m^3^, it is difficult to draw direct conclusions on the relative toxicity of the two materials using information on the lung deposition. However, this information does facilitate comparison with other studies.

It has been suggested that inflammation following inhalation of nanomaterials (e.g., expressed in terms of % neutrophils in BALF) is driven by deposited surface area doses [17,47]. For example, an analysis of results from a number of studies covering a wide range of nanomaterials indicated that the onset surface area dose for 6% neutrophils for a range of CNT (MWNT-7, NM-401, NM-403, and Nanocyl NC7000) was between 3 and 5 cm^2^ [48]. Our results for pMWCNT (NM-401) are consistent with these results, as at surface area doses of 0.5 and 1 cm^2^, no increase in neutrophils beyond background was seen. However, for MWCNT-COOH, no increase was seen in neutrophils at much larger surface area doses of 208 cm^2^ and 595 cm^2^. This suggests that MWCNT-COOH are less inflammogenic than pMWCNT, consistent with other studies that have indicated that carboxylated MWCNT tend to exhibit lower toxicity [19,49,50]. The study exploring the link between deposited surface area and neutrophil influx [48] also identified a set of two CNTs (Baytubes and JC 162) which were much less inflammogenic than the other set, with an onset surface area for 6% neutrophils for Baytubes of 130 cm^2^ and >200 cm^2^ for JC 162. It appears that MWCNT-COOH may behave more similarly to this group of MWCNTs. MWCNT-COOH, Baytubes, and JC 162 are all narrow fibres (diameter ≤ 10 nm) which, when aerosolised, produce highly agglomerated aerosol particles. In particular, Baytube aerosols have a similar morphology (sphere-like agglomerates) and size (MMAD 1.7–2.2 µm) to the MWCNT-COOH aerosol. The lower toxicity of MWCNT-COOH in comparison to pMWCNT may, therefore, also be explained by the difference in lung-deposited aerosol particle morphologies, i.e., more rigid (thicker) MWCNTs are more toxic than less rigid fibres, which produce highly agglomerated aerosol particles. This is consistent with the results of in vivo toxicity studies on MWCNTs introduced via pharyngeal aspiration, which showed that long tangled MWCNTs with low rigidity appeared less toxic than long rod-like MWCNTs with high rigidity (e.g., MWCNT-7) [10,51,52].

### 4.3. Comparison of Whole-Body and Nose-Only Exposure Studies of pMWCNT (NM-401)

In a study carried out to compare the pulmonary toxicity of two types of MWCNTs delivered by intratracheal instillation vs. nose-only inhalation, Gaté et al. [22] undertook a nose-only inhalation study using one of the materials we investigated, pMWCNT (NM-401), using the same exposure concentrations (0.5 and 1.5 mg/m^3^), MWCNT aerosolization system (acoustic generator), animal model (female Sprague–Dawley rats), and exposure duration (6 h/day × 5 days/week × 4 weeks) as those used here. Both studies found no significant differences in pMWCNT-exposed H&E-stained tissues in comparison to control, other than the presence of pigmented cells, and no significant differences in the amount and distribution of collagen were observed using Masson’s Trichrome stain in either. However, despite the similarities in the experimental design and these negative histopathology results, the nose-only exposure study resulted in greater BALF cytotoxicity and inflammatory responses than our whole-body study. For example, our whole-body study found no significant changes in LDH or total protein levels, whereas the nose-only study found a dose-response-related increase in both at day 3. Similarly, our study indicated no change in BALF total cell, macrophage, or neutrophil numbers but Gaté et al. [22] found significant increases in neutrophil numbers at the 1.5 mg/m^3^ level, which reduced with time post-exposure, and significant changes in total cell and macrophage numbers, albeit with a less clear dose and time response. It is hypothesised that this difference in outcomes is a result of differences in particle deposition within the lung between the two studies, a consequence of differences in aerosol characteristics and/or animal behaviour and breathing patterns.

**Figure 9:**
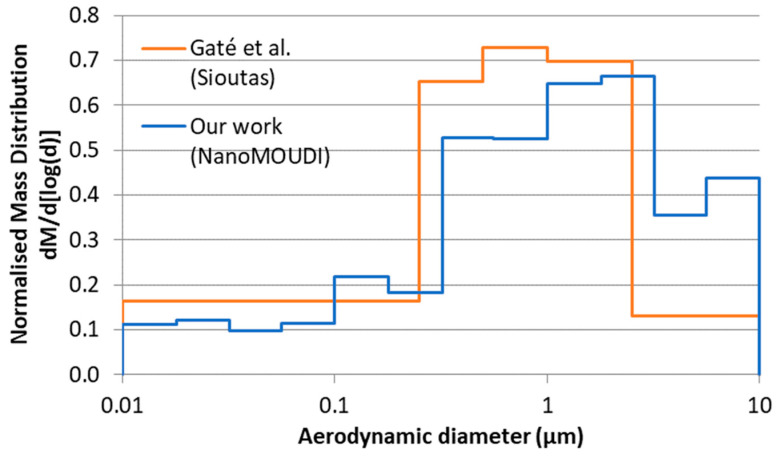
Comparison of normalised pMWCNT aerosol mass size distribution from this work (average of 3 measurements using NanoMOUDI) with that reported in [22].

Both studies used MPPD to estimate deposition fractions and total deposited masses (see Table 5 for a comparison). Estimated deposition fractions in the head were similar in both studies, however, deposition fractions in the TB and pulmonary region were 2–3 times and 100–600 times higher, respectively, in Gaté et al. [22] than our study, resulting in an estimated pulmonary deposition of 6 µg here compared to 240 µg for 1.5 mg/m^3^. In both studies, an aspect ratio of 30 was assumed; however, the effective density adopted differed significantly (0.36 g/cm^3^ cf 0.032 g/cm^3^). Effective densities are complex to determine [31,53,54,55]; however, if we had used the same effective density, this would have increased estimated pulmonary deposition by a factor of approximately 5 to 30 µg (see Appendix A), still lower by a factor of 8 than Gaté et al. [22]. It is, therefore, likely that other differences in the aerosol characteristics are driving differences in deposition estimates between the two studies. Notably, the sizes of the aerosols produced in our study were larger than those produced in [22]. Figure 9 shows the aerosol mass size distribution for our work (average of 3 measurements using NanoMOUDI, normalised to total mass reported by Gaté et al. [22]) compared with the data reported by Gaté et al. [22] using Sioutas Cascade Impactor. It is clear that in our work, a significant number of larger (>3 µm) particles were produced during the generation process, which were less apparent in [22]. This is also evident by comparison of the higher MMAD observed in our measurements (1.57 µm and 2.01 µm for low and medium target concentrations, respectively, Table 1) compared with 0.79 µm in [22]. These particles are in sizes where the inhalability fraction of particles by rats begins to decrease [56]. Although this is accounted for in our MPPD calculations, in practice it means that, for a given target mass concentration, proportionally less of the material is likely to reach and deposit in the lungs, potentially contributing to the lower estimated mass and surface area deposition in our study compared to [22] even though our observed airborne mass concentrations were similar. This is an important finding, as both studies were in many respects very similar, but the results reflect the fact that small differences in system design can have a marked effect on aerosol characteristics and, thus, biological endpoints.

In addition to differences in aerosol characteristics, it is possible that other factors, including breathing rates, could impact deposition levels. Both studies used the default MPPD breathing parameters for the specific exposure mode modified by rat mass (i.e., the default of 295 g was used for all except nose-only for 0.5 mg/m^3^, which assumed 415 g). For example, Miller et al. [57] reviewed the literature and reported a 38% higher breathing rate in rats restrained in nose-only tubes compared to whole-body breathing, considered to be due to the higher stress level. Simplistically, it might be inferred that a lower breathing rate would lead to lower deposition, it is, however, important to note that some comparison studies have identified no statistically significant effect of exposure modality on lung deposition [58] and/or identified higher lung deposition using whole-body chambers [59]. The relationship between breathing rate and deposition is complex. For example, in the sensitivity analysis reported in Appendix A, using MPPD we show that reducing the breathing rate by 20% has no impact on pulmonary deposition but increases thoracic deposition by 10%, and that increasing the breathing rate by 40% and assuming nose-only exposure reduces pulmonary and thoracic deposition by between 10 and 20%. It is thus unlikely that differences in deposition due to breathing patterns are contributing significantly to the differences between the results of the two studies, although clearly, a sensitivity analysis that considers the separate effects of different parameters cannot explore the full range of uncertainty.

Another difference between whole-body and nose-only systems is that the animals typically have more freedom of movement in the former. It is, thus, possible for animals to lie such that their breathing zone is partly protected by fur, which may act as a particle filter. This is difficult to reflect in a model but may have a significant impact on actual levels of deposition.

Measurements of lung burden would do much to simplify such comparisons, clarify the source of such differences, and allow clearer comparison between studies, although it is accepted that this can be challenging for MWCNTs [45,46]. It is also important to reflect that typical model estimates of the deposition of fibrous material aerosols are considered significantly more uncertain than those for other aerosol types [31], with some authors opining that ‘currently the use of computational models of deposition are not considered sufficiently robust to predict the deposition of fibre shaped materials’ [27]. Given the complexity of the aerosols that can be produced using such materials (e.g., Figure 2, pMWCNT), with subgroups of the aerosol with different aspect ratios and effective densities, choosing single parameter values to represent the complete aerosol will remain challenging, and further work is needed to improve these models.

### 4.4. MWCNT-COOH and pMWCNT Have Different Effects on Global Gene Expression and Selected Cytokines

Despite no significant adverse histopathological effects being observed, transcriptomic analysis indicated some changes in gene expression resulting from the exposures at 1.5 mg/m^3^, with differences seen between the two materials (Figure 6), i.e., fewer differentially expressed genes (DEGs) for the MWCNT-COOH (49) than pMWCNT (76) and, unexpectedly, no overlapping DEGs (Supplementary Information Figure S7). When analysing the DEGs in lung samples 3 days post-exposure to pMWCNTs, the 76 genes were used for Ingenuity Pathway Analysis (IPA). The top three canonical pathways identified were immune and inflammatory responses, including agranulocyte adhesion and diapedesis, granulocyte adhesion and diapedesis, and acute phase response signalling. These results are in agreement with previous findings following intratracheal instillation of mice using a panel of CNT [60,61].

Additionally, in this study, both mRNA levels from RNA-seq and protein levels from ELISA demonstrated significant upregulation of Cxcl1 and OPN in lung tissues and BALF following exposure to pMWCNT (Figure 7). This suggests that immune and inflammatory pathways likely contribute to the pulmonary responses observed. The gene expression pattern following exposure to MWCNT-COOH was much less clear (Supplementary Information Figure S7), at a level typical of noise, and there was no significant change in Cxcl1 and OPN. These results again suggest that the toxicity of pMWCNT is greater than that of MWCNT-COOH.

Some of the DEGs identified in the current study have been observed in other animal studies involving exposure to CNTs. For example, in a whole-body exposure study on MWCNT-7, Sager et al. [62] observed that the upregulation of all tested cytokines, including IL-1β, was dose-dependent based on the amount of MWCNT-7 inhaled by the rats. In addition, Fujita et al. found several genes, including *Ccl3*, *Ccl6*, and *Ccl9*, were upregulated at 1 day post-instillation of SWCNTs [63]. Of particular relevance to this study, Seidel et al. [23] analysed the lung tissue transcriptome of animals exposed to NM-401 (pMWCNT) and NM-403 through nose-only inhalation [22], and as in this study, found significant increased expression of SPP1 on day 3 after exposure to 1.5 mg/m^3^ of NM-401 and also in-creases in Ccl3 and Ccl9. In comparison to our study, they identified a greater number of DEGs (1256) following exposure to pMWCNT, which again supports the above argument that, despite many similarities in study design, the lung deposited dose was lower in our study.

### 4.5. Lung Fibrogenic Effects Induced by CNTs and the Relevance of Osteopontin

Several in vivo studies in the literature have demonstrated that the inhalation of CNTs can induce fibrogenic effects in the lungs [64,65,66,67,68,69,70,71]. Osteopontin (OPN), a glycoprotein secreted by various cell types, including inflammatory, immune, fibroblast, osteoblast, and cancer cells, is believed to play a key role in CNT-induced lung fibrosis [35]. T-helper 2 (Th2)-dependent immune pathways are recognised as key drivers in promoting CNT-induced lung fibrosis by producing type 2 pro-fibrotic factors, including OPN [72]. Single-walled carbon nanotubes (SWCNTs) have also been shown to induce fibrogenic responses in the lungs, characterised by OPN upregulation, which subsequently stimulates TGF-β1 expression and activation, promoting fibroblast-to-myofibroblast differentiation [73]. Additionally, pathways such as the TIMP1/CD63/integrin β1 axis and ERK signaling have been implicated in MWCNT-induced lung fibrosis [74].

Although a direct molecular mechanism linking CNTs to lung fibrosis remains unidentified, our previous studies using other nanoparticles (Ag [75] and CeO_2_ [76]) in nose-only inhalation experiments with Sprague–Dawley rats also showed significant upregulation of OPN in bronchoalveolar lavage fluid (BALF), similar to our current findings. While pMWCNT-induced lung pathology did not show significant fibrosis (Table 4, Supplementary Information Figure S5), OPN expression in alveolar macrophages was significantly elevated in response to internalised pMWCNT agglomerates (Figure 8), suggesting potential pre-fibrotic pulmonary effects, particularly chronic and terminal outcomes, may originate in alveolar macrophages.

### 4.6. Lung Clearance

A semi-quantitative analysis of lung clearance based on pigmented macrophages indicated a similar initial clearance pattern for both MWCNTs with a retention half-time of approximately 30–40 days (Supplementary Information Figure S6). This is broadly consistent with reported lung retention half-times in rats following the inhalation of low concentrations of low solubility nanoparticles (e.g., TiO_2_, carbon black, CeO_2_) in the range of 40–60 days [77,78,79]. Kim et al. [39] found an initial retention half-time of about 35 days for tangled MWCNTs following 28 days of exposure, and Ellinger-Ziegebauer and Pauluhn [80] an initial half-time of 60 days following a 6 h exposure to 11 mg/m^3^ of a tangled MWCNT (Baytubes^TM^) aerosol, which are both broadly consistent with the results here. Some other studies have found slower clearance of inhaled MWCNTs; however, these typically reflect varying degrees of clearance inhibition due to overload conditions [42].

It is important to note that particle clearance patterns are not monotonic, and clearance typically slows over time post-exposure. Our identification of MWCNT agglomerates and fibres in tissue sections at 1 year post-exposure is consistent with the presence of these slower longer-term clearance pathways. MWCNT-COOH were present as agglomerates and pMWCNT mainly as single fibres, which is consistent with the findings from an intratracheal instillation study using 11 CNTs, which found at 1 year that short and thin MWCNTs were observed as agglomerates but longer and thicker MWCNTs, including NM-401, were seen as single fibres throughout the lung [18].

Mercer et al. [65] importantly identified different clearance rates for inhaled aerosol particles with varying morphologies, with large agglomerates (>4 fibres) accounting for the majority of clearance and single fibres not significantly cleared from the lung over 168 days post-exposure. This long-term presence of small numbers of single fibres may be consistent with that seen here for pMWCNT using enhanced darkfield microscopy. However, further detailed studies would be needed to confirm this and any overall differences in clearance between the two materials.

It is important to reflect that clearance from the human lung is typically slower than from rodents. Kuempel et al. [81] developed a model of inhaled particle retention in the alveolar region based on data from coal miners, which comprises an alveolar compartment that clears to both the tracheobronchial region and the alveolar interstitium, with the particles in the interstitium clearing slowly to lymph nodes. A more recent model was developed using additional human data from other studies [82]. This found the best fit to experimental data using particle transfer rates following deposition of insoluble particles in the alveolar region of 0.002 d^−1^ to the tracheobronchial region and 0.001 d^−1^ to the alveolar interstitium, giving a clearance half-time of approximately 250 days, with approximately 33% of the alveolar deposition of insoluble particle sequestered in the interstitium, with a clearance rate to lymph nodes of 10^−5^ d^−1^ (equivalent to a clearance half-time > 100 years). Care therefore needs to be taken when using the results of lung clearance studies in rodents in relation to human health hazard and risk assessments.

### 4.7. Relevance of MWCNT Exposures

This study used aerosol concentrations in the range 0.5–4.5 mg/m^3^, which are significantly higher than those typically measured within occupational settings. For example, a review of occupational exposures to nanomaterials identified 52 MWCNT exposure situations with quantitative measurements, with elemental carbon (EC) concentrations in the workers’ breathing zone ranging from <0.5 µg/m^3^ to 48 µg/m^3^ and aerosol particle counts ranging from 0.002 to 200 per cm^3^ [83]. A later assessment of carbon nanotube and nanofibre workers at 12 facilities across the US using personal air samplers found that the mean daily exposure to EC was 1.0 µg/m^3^ in the respirable fraction (range 0–44 µg/m^3^) and 6.2 µg/m^3^ in the inhalable fraction (range 0–418 µg/m^3^) with a mean exposure of 0.13 CNT/F structures/cm^3^ estimated from TEM images [84]. However, an exposure of 28 days was used here rather than the chronic exposures that may occur within the working environment. In terms of exposure, the 1.5 mg/m^3^ used in this study is broadly equivalent to 0.15 mg/m^3^ for a working year and 4 µg/m^3^ for a 40-year working lifetime, again at the upper end of occupational exposure levels and above the US NIOSH recommended 8-h exposure limit for CNT of 1 μg/m^3^ of respirable EC [85]. Exposure by consumers is considered unlikely as CNT are typically present within composites, with modelled exposures < ng/m^3^ [86].

## 5. Conclusions

The primary objective of this study is to explore the effect of differences in physicochemical properties of MWCNT on acute and chronic pulmonary effects. To this end, two different MWCNTs were assessed using a 28-day inhalation exposure protocol, following the animals for 1 year post-exposure. The two materials chosen were a ‘straight’ long (4 µm) and thick (70 nm) pristine MWCNT (pMWCNT) and a chemically functionalized (COOH, 4 wt%), ‘tangled’, very long (10–30 µm), and thin (<8 nm) MWCNT (MWCNT-COOH).

At the aerosol concentrations used, only low levels of biological effects were seen (e.g., no significant histopathological changes to 1 year). As the concentrations used were above measured occupational exposure levels and exposure limits, this is a positive result in terms of the indication of potential risk; however, the low levels of biological effect inhibited a detailed comparison of the two materials. This was further complicated by the difficulties in aerosolising pMWCNT, which limited the cross-material comparison to a single concentration of 1.5 mg/m^3^, a level at which BALF cytology and biochemistry (LDH, total protein, ALP) indicated no significant differences between the materials and controls.

Transcriptomic analysis did, however, indicate some changes in gene expression at 1.5 mg/m^3^ at 3 days with clear differences between the two materials. The DEGs induced by pMWCNT could be broadly characterised as being involved in pro-inflammatory processes, whereas the gene expression pattern following MWCNT-COOH exposure was less clear at a level typical of noise. BALF analysis of several cytokines and chemokines relevant to pro-inflammatory and fibrotic responses (IL-1β, Cxcl1, Ccl2 (MCP-1) and osteopontin) indicated no difference from controls for MWCNT-COOH, whilst for pMWCNT significant differences for Cxcl1 and osteopontin were seen. This difference was also seen in immunofluorescence staining for osteopontin in lung tissues, suggesting pre-fibrotic processes. These results indicate that pMWCNT had a greater effect than MWCNT-COOH at the same aerosol concentration level; however, toxicological effects are clearly driven by the levels of deposited dose, and considering these, the difference between the materials became more marked. Although the aerosol particle sizes used (MMAD 1.6–2.2 µm, GSD 1.5–2.0) complied with OECD guidance [43,44], designed to ensure sufficient exposure of the lower respiratory tract (i.e., MMAD ≤ 2 µm, GSD 1–3), MPPD generated estimates of deposition of both materials were low, particularly for pMWCNT. These revealed that MWCNT-COOH was more effectively deposited than pMWCNT, by a factor of 7 for mass and 200 for surface area, thus reinforcing that pMWCNT was clearly more toxic than MWCNT-COOH.

Unfortunately, the low levels of deposited dose and resulting limited effects on gene expression, especially for MWCNT-COOH, meant that a detailed comparison of underlying molecular mechanisms that could potentially have elucidated differences arising from the differences in physicochemical characteristics was not possible. The results tend to confirm findings from previous studies that straight thick fibres, even those at lengths below which frustrated phagocytosis would be expected, result in greater effects than long and thin tangled fibres. Further studies with an enhanced design resulting in greater deposited doses and a wider panel of materials are required to improve understanding in this area.

Comparison with the results from a very similar study using pMWCNT revealed that even small differences in experimental system design can have a marked effect on lung deposition and thus biological endpoints, further emphasising the need to understand deposited doses when assessing the results of inhalation studies with nanomaterials.

Although the biological effects seen here were limited, even at levels well above occupational exposure levels, thus suggesting potentially low risks, imaging indicated that particles were present in the lung at 1 year post-exposure. This is a potential concern for chronic exposure situations, especially given the generally slower clearance of particles from human than rodent lungs, and an issue worthy of further exploration.

## Figures and Tables

**Table 1:** Physiochemical characteristics of the MWCNT.

MWCNT	Diameter (nm)	Length (µm)	Specific Surface Area (m^2^/g)	COOH (%wt)
pMWCNT ^(a)^	67 ± 24	4.0 ± 2.3	18	NA *
MWCNT-COOH ^(b)^	<8	10–30	500	3.86

^(a)^ data from [29] ^(b)^ data from supplier, JRC. * Not available.

**Table 2:** MWCNT Aerosol characteristics.

MWCNT	Target Conc. (mg/m^3^)	Gravimetric Mass Conc. (mg/m^3^)	APS					CPC
			CMAD (µm)	GSD (CMAD)	MMAD (µm)	GSD (MMAD)	No. Conc. (particles/cm^3^)	No. Conc. (particles/cm^3^)
pMWCNT	0.5	0.54 ± 0.04	0.80 ± 0.01	1.44	1.57 ± 0.07	1.65	190 ± 13	673 ± 79
	1.5	1.50 ± 0.45	0.79 ± 0.02	1.48	2.01 ± 0.34	1.95	469 ± 106	1520 ± 328
MWCNT-COOH	1.5	1.71 ± 0.23	1.35 ± 0.02	1.52	2.15 ± 0.03	1.45	187 ± 17	195 ± 21
	4.5	4.48 ± 1.09	1.24 ± 0.05	1.55	2.17 ± 0.06	1.51	537 ± 107	392 ± 69

APS, aerodynamic particle sizer; CPC, condensation particle counter; CMAD, count median aerodynamic diameter; GSD, geometric standard deviation; MMAD, mass median aerodynamic diameter.

**Table 3:** Estimates of deposition fractions, mass, and surface area deposited doses in the respiratory tract determined using MPPD.

MWCNT	Effective Density (g/cm^3^)	Target Conc. (mg/m^3^)	Deposition Fraction			Thoracic Deposition (µg)	Pulmonary Deposition (µg)	Pulmonary Surface Area Deposition (cm^2^)
			**H**	**TB**	**P**			
pMWCNT	0.032	0.5 (Low)	0.349	0.057	0.0004	54.2	3.0	0.5
		1.5 (Medium)	0.268	0.042	0.0001	109.2	6.1	1.1
MWCNT-COOH	0.071	1.5 (Medium)	0.385	0.020	0.014	101.5	41.5	207.6
		4.5 (High)	0.381	0.021	0.016	288.1	119.0	595.2

H, head; TB, tracheobronchial; P, pulmonary.

**Table 4:** Severity scoring of histopathological effects in lung sections from rats exposed to two types of MWCNT aerosols (1.5 mg/m^3^) at 3 days and 12 months post-exposure.

Time	Material	Histopathological Characteristic *							
		Alv Oed	Alv M	M Pig	Alv Fibr	Br CC Prolif	Br Loss Cilia	Int Oed	Int Fibr
3 days	Air (n = 5)	0.00	0.40	0.00	0.00	0.00	0.00	0.00	0.00
	pMWCNT (n = 6)	0.00	1.50	1.83	0.00	0.00	0.00	0.00	0.00
	MWCNT-COOH (n = 5)	0.00	1.40	2.40	0.00	0.00	0.00	0.00	0.00
1 year	Air (n = 4)	0.00	0.75	0.00	0.00	0.00	0.00	0.00	0.00
	pMWCNT (n = 6)	0.00	1.67	0.33	0.00	0.00	0.00	0.00	0.00
	MWCNT-COOH (n = 6)	0.00	0.83	0.00	0.00	0.00	0.17	0.00	0.00

* Alv oed, alveolar oedema; Alv M, alveolar macrophages; M pig, pigmented macrophages; Alv fibr, alveolar fibrosis; Br CC prolif, club cell proliferation; Br loss cilia, loss of cilia; Int oed, interstitial oedema; Int fibr, interstitial fibrosis. Scoring system: 0 = none, 1 = minimal, 2 = mild, 3 = moderate, 4 = marked/severe.

**Table 5:** Comparison of aerosol parameters and modelled estimates of deposition fractions, mass and surface area deposited doses in the respiratory tract for 28-day whole-body (this study) and nose-only (Gaté et al. [22]) inhalation studies with pMWCNT (NM-401).

Exposure	Effective Density (g/cm^3^)	Mass Conc. (mg/m^3^)	MMAD (µm)	Deposition Fraction			T Deposition (µg)	P Deposition (µg)	P Deposition (cm^2^)
				H	TB	P			
Whole-body *	0.032	0.54 ± 0.04	1.57	0.349	0.057	0.0004	54	3.0	0.5
		1.50 ± 0.45	2.01	0.268	0.042	0.0001	109	6.1	1.1
Nose-only ^#^	0.36	0.54 ± 0.11	0.79	0.318	0.095	0.045	279	90	16
		1.49 ± 0.24	0.79	0.304	0.119	0.060	709	239	43

H, head; TB, tracheobronchial; T, thoracic; P, pulmonary; * this study; ^#^ [22].

## Data Availability

The original contributions presented in the study are included in the article/Supplementary Material; further inquiries can be directed to the corresponding authors.

## Supplementary Materials

The following supporting information can be downloaded at https://www.mdpi.com/article/10.3390/toxics13050401/s1: Figure S1. Experimental aerosol exposure set-up: (A) schematic diagram of the experimental setup (MFC: mass flow controller, HEPA: high-efficiency particulate air filter, SMPS: scanning mobility particle sizer), (B,C) photographs of aerosol generator; Figure S2. Representative TEM images of MWCNT aerosol particles; Figure S3. Cytological analysis of bronchoalveolar lavage fluid: (A) Total cell counts; (B) Distribution of macrophages; (C) Distribution of neutrophils; Figure S4. Cytotoxicity analysis of bronchoalveolar lavage fluid. (A) Relative total protein (TP) levels; (B) Relative alkaline phosphatase (ALP) levels; Figure S5. Trichrome-Masson stained lung sections from rats exposed to two types of MWCNT aerosols at 3 days and 1 year post-exposure; Figure S6. Localisation of inhaled MWCNT particles in macrophages recovered by bronchoalveolar lavage from rats at 3 days, 30 days, and 1 year post-exposure to two types of MWCNT aerosols at the medium aerosol concentration: (A) representative brightfield microscope images of recovered macrophages and (B) percentage of macrophages with observed pigments (agglomerates of MWCNTs) recovered from bronchoalveolar lavage; Figure S7. Venn diagram of significant differentially expressed genes (DEGs) compared between two types of MWCNT aerosols, with significant differential expression in lung tissues from rats exposed to MWCNT-COOH or pMWCNT (NM401) at 3 days post-exposure; Figure S8. Expression of cytokines Cxcl1 and OPN, in protein levels in BALF from rats at 3 days, 30 days, and 1 year post-exposure to the medium aerosol concentration of two types of MWCNT aerosols relative to the unexposed controls at different time points post-exposure; Figure S9. Regional deposition pattern for each MWCNT (A,B = MWCNT-COOH, C,D = pMWCNT) for target concentration 1.5 mg m^−3^ showing the effect of altering aspect ratio (A,C) and effective density (B,D) on computed MPPD results; Table S1. Elemental impurities detected in MWNCTs using ICP-MS; Table S2. Aerosol parameters for each mode obtained from fitting bimodal log-normal distributions to mass size distributions derived from APS results and Table S3. Pulmonary distribution of MWCNTs from enhanced darkfield microscopy images—incidence table.

## Abbreviations

The following abbreviations are used in this manuscript:

APS	Aerodynamic particle sizer
AR	Aspect ratio
BAL	Bronchoalveolar lavage
BALF	Bronchoalveolar lavage fluid
CF	Carbon nanofibre
CMAD	Count median aerodynamic diameter
CNT	Carbon nanotubes
CPC	Condensation particle counter
DEG	Differentially expressed gene
GSD	Geometric standard deviation
H&E	Hematoxylin&Eosin
HARN	High aspect ratio nanomaterial
IATA	Integrated approach to testing and assessment
LDH	Lactate dehydrogenase
MMAD	Mass median aerodynamic diameter
MWCNT	Multi-walled carbon nanotube
NOAEL	No observed adverse effect level
OPN	Osteopontin
PCA	Principal component analysis
SWCNT	Single-walled carbon nanotube
TB	Tracheobronchial
TEM	Transmission electron microscopy
TP	Total protein

## Appendix A

Estimating deposition within the respiratory tract and its component parts using the MPPD model requires assumptions to be made about a large number of parameter values, including those relating to the aerosol and the animal model. Many of these have a significant degree of associated uncertainty. To explore the effect on deposited dose estimates of variations in a number of key parameters [31] (e.g., breathing rates, aerosol particle effective density, and aspect ratio), a limited sensitivity analysis was undertaken to support the discussion and interpretation of the study results.

Aerosol density is a required input in MPPD and is also needed to convert between aerosol aerodynamic diameter and mass. The aerosol effective density is often not the same as either the ‘bulk’ or absolute densities of the material [87]. The absolute densities of the related materials graphite and carbon black are between 1.8 and 2.2 g/cm^3^ and the density of individual CNT fibres have been reported as being in the range of 1.7–2.2 g/cm^3^; however, broadly spherical agglomerates may have significantly lower effective densities in the range of 0.1–1.0 g/cm^3^, with some studies estimating even lower densities, e.g., 0.01 g/cm^3^ (see [31] for a recent discussion of CNT aerosol effective densities). Thus, aerosol effective densities can vary over 2 orders of magnitude. Aerosol effective density is not straightforward to derive [31]. In this study, it was estimated using the APS aerosol data and the gravimetric mass concentration, i.e., the assumed density in the APS software, was changed to satisfy the condition that the total mass concentration matched the observed gravimetric mass. This approach resulted in estimated effective densities of 0.032 g/cm^3^ for pMWCNT (similar to the reported bulk density of 0.02 g/cm^3^ [29]) and 0.071 g/cm^3^ for MWCNT-COOH, both toward the lower end of the observed range in the literature.

In the case of pMWCNT (where a direct comparison with [22] is possible as they used the same material), this is significantly lower than previous estimates. This could be due to several reasons, including APS instrument performance against non-unit-density aero-sol, but also the presence of particles in the gravimetric sample which were not within the size range of the APS. In particular, we note that Stage 1 of the NanoMOUDI (intended as a ‘filter’ for particles larger than 10 µm) collected a number of visibly large aggregates which were likely to have been >20 µm, i.e., would not have been detected by APS but would have contributed to measured gravimetric mass. Ignoring the Stage 1 masses, approximate effective densities of 0.28 g/cm^3^ for MWCNT-COOH and 0.07–0.14 g/cm^3^ for pMWCNT can be estimated, which are higher than the base case assumptions; however, as mentioned previously, the limited number of measurements made means that there is some uncertainty on these values.

In light of the uncertainty surrounding effective density estimation, we therefore undertook MPPD calculations for a range of effective densities to investigate how this may have affected estimated deposited doses. For pMWCNT, in addition to our estimated density of 0.032 g/cm^3^, we also used the estimated density for an aerosol produced using the same material from a previous published study, 0.36 g/cm^3^ [22], and a density of 1 g/cm^3^, a value approaching the maximum generally seen. For MWCNT-COOH, in addition to the estimated value of 0.071 g/cm^3^, we chose a density of 0.4 g/cm^3^ based on a morphologically similar MWCNT, Flotube 9000 [87], and a higher density of 0.8 g/cm^3^, again reflecting the higher end of the range, to illustrate trends in deposition patterns. All other parameter values used in the MPPD model remained the same as for the base case, except that the base case MMD was assumed to be the same as the MMAD. It is accepted that this is a simplification, but as the objective was to explore the relative effect of differences in density, this was considered appropriate. Clearly a full uncertainty analysis exploring the ranges in all parameters simultaneously would provide a more robust indication of the range of deposition, but was beyond the scope of this study.

For both materials, increases in effective density by up to a factor of 30 have a minimal effect on deposition fraction within the head airways (Table A1). Estimated thoracic deposition doses for pMWCNT also show minimal change (+10%). For MWCNT-COOH, overall total thoracic deposition decreases as assumed effective density increases, by up to 40%. However, when considering pulmonary deposition, for MWCNT-COOH, there is a decrease (with increasing density) that mirrors total thoracic deposition, while for pMWCNT, pulmonary deposition increases markedly with increasing density (i.e., increasing density by a factor of 30 increases deposition by a factor of 8). This is apparent when examining the deposition across different lung generations, shown in Supplementary Information Figure S9, where increasing the density ‘shifts’ the deposition of pMWCNT to the deeper lung.

**Table A1:** Estimates of deposition fraction and thoracic mass, pulmonary mass, and pulmonary surface area deposited MWCNT doses for different effective densities for an aerosol concentration of 1.5 mg m^−3^, determined using MPPD.

MWCNT	Effective Density (g/cm^3^)	H Fraction	TB Fraction	P Fraction	Thoracic Deposition (µg)	Pulmonary Deposition (µg)	Pulmonary Deposition (cm^2^)
pMWCNT	**0.032 ***	**0.268**	**0.042**	**0.0001**	**109.2 (1.0)** ^+^	**6.1 (1.0)** ^+^	**1.1**
	0.36	0.267	0.034	0.011	115.6 (1.1) ^+^	33.3 (5.5) ^+^	6.0
	1.0	0.271	0.027	0.018	117.8 (1.1) ^+^	46.4 (7.6) ^+^	8.4
MWCNT-COOH	**0.071 ***	**0.385**	**0.020**	**0.014**	**101.5 (1.0)** ^+^	**41.5 (1.0)** ^+^	**207.6**
	0.4	0.400	0.016	0.010	76.2 (0.8) ^+^	28.7 (0.7) ^+^	143.7
	0.8	0.404	0.015	0.007	65.7 (0.6) ^+^	21.2 (0.5) ^+^	105.9

* base case assumptions in bold text (Table 2), ^+^ ratio of variant to base case.

Aspect ratio (AR) plays an important role in determining respiratory system deposition, in particular by interception [28,31]. TEM images indicated MWCNT-COOH aerosols as agglomerates, broadly spherical in shape for all particle size ranges (Figure 2 and Supplementary Information Figure S2), with aspect ratios generally in the range of 1 to 4. To determine deposition, a base case AR of 4 was used, and to explore the effect of changes in AR, values of 1 and 8, effectively bounding the range of observed AR, were used in the sensitivity analysis; all other parameter values remained the same as for the base case. The pMWCNT aerosol was much more complex, with single fibres of various diameters, complex fibre bundles, and broadly spherical particles (Figure 2 and Supplementary Information Figure S2). Aspect ratios ranged from 1 for spherical agglomerates to approximately 150 for the longer single fibres. Choosing a single AR to reflect the deposition behaviour of this aerosol is not straightforward. For the base case deposition calculations, an aspect ratio of 30 was used, which was intended to broadly reflect the mixture with a greater weighting for the lower AR particles, which are considered more reflective of the (mass-weighted) average. This value is also consistent with that used in a previous study with the same material [22]. However, this value clearly has significant uncertainty. Ideally, a deposition model would allow different AR values for subcomponents of the aerosol to allow for this variation. For the sensitivity analysis, lower aspect ratios of 1, 8, and 15 were used, and one higher value of 60 (this is the ratio of average length to average diameter [29]). These are intended to broadly bound the effective range; all other parameter values used in the MPPD model remained the same as for the base case.

The results (Table A2) indicate that for both MWCNT types, increasing the AR reduces the deposition fractions in the head airways and pulmonary region. The thoracic deposition fraction increases from an AR of 1 to 8, but then follows the same pattern of reduction with increasing AR. These differences are also reflected in the deposited doses. For MWCNT-COOH, the changes in thoracic and pulmonary deposited mass doses are similar, with a maximum of 1.7 times the base case (AR = 4) dose at an AR of 1 and 0.4–0.6 times the base value at an AR of 8. For pMWCNT, the changes in the pulmonary deposited dose are much greater than for the thoracic deposited dose. The thoracic dose varies between a factor of 1.6 and 0.8 of the base case value, whereas the pulmonary dose range spans two orders of magnitude (i.e., 10–0.1 times the base value). Examining the deposition across different lung generations (Supplementary Information Figure S9), immediately obvious is a peak at airway generation 5 for all AR ≥ 4. Beyond this, again we observe a shift for pMWCNT, in this case to higher generations in the lung as AR increases, as well as an overall decrease in deposition. Also of note is that deposition for pMWCNT for AR = 4 is greater than for AR = 1, driven by additional deposition in Generations ~8–22.

**Table A2:** Estimates of deposition fractions and thoracic mass, pulmonary mass, and pulmonary surface area deposited MWCNT doses at different aspect ratios for an aerosol concentration of 1.5 mg m^−3^, determined using MPPD.

MWCNT	Aspect Ratio	H Fraction	TB Fraction	P Fraction	Thoracic Deposition (µg)	Pulmonary Deposition (µg)	Pulmonary Deposition (cm^2^)
pMWCNT	1	0.429	0.033	0.022	144.2 (1.3) ^+^	62.7 *(10.3) ^+^*	11.3
	8	0.290	0.051	0.017	177.3 (1.6) ^+^	52.2 (8.6) ^+^	9.4
	15	0.280	0.050	0.003	138.7 (1.3) ^+^	22.5 (3.7) ^+^	4.1
	**30 ***	**0.268**	**0.042**	**0.0001**	**109.2 (1.0) ^+^**	**6.1 (1.0)** ^+^	**1.1**
	60	0.257	0.034	<0.0001	89.1 (0.8) ^+^	0.7 (0.1) ^+^	0.1
MWCNT-COOH	1	0.657	0.037	0.021	171.5 (1.7) ^+^	69.1 (1.7) ^+^	345.3
	**4 ***	**0.385**	**0.020**	**0.014**	**101.5 (1.0) ^+^**	**41.5 (1.0)** ^+^	**207.6**
	8	0.346	0.014	0.005	57.0 (0.6) ^+^	17.3 (0.4) ^+^	86.7

* base case assumptions in bold text (Table 2), ^+^ ratio of variant to base case.

For the base case calculations of deposition, we used the MPPD whole-body exposure mode option and the associated default breathing parameters for a default rat mass of 295 g. To compare our results with those from a similar study using pMWCNT but in a nose-only exposure system [22], we also estimated deposition using MPPD with the nose-only exposure mode option with default parameters for that option. As animals were often observed to be asleep during exposures, we also investigated the effect of a potentially lower breathing frequency using the whole-body exposure mode option with all other breathing parameters as default. A breathing rate 20% lower than the default was chosen, which is consistent with studies that have found breathing rates and minute ventilation levels 20–30% [88] and 4–30% [89] lower during sleeping. All other parameter values used in the MPPD model remained the same as for the base case. In all cases, deposition is lower (Table A3) with the assumption of nose-only breathing compared with whole body, and a lower breathing frequency results in a higher estimated deposition, despite overall volume breathed being lower. This is possibly because, for whole-body (c.f. nose-only) and lower breathing frequency, residence time within the lung is longer, meaning that deposition efficiency via sedimentation (which is expected to be significant for these particle sizes) could be increased. The differences in deposited mass in relation to the base case for pMWCNT were <10% for pulmonary and <20% for thoracic. The differences were greater for MWCNT-COOH.

**Table A3:** Estimates of thoracic mass, pulmonary mass, and pulmonary surface area deposited MWCNT doses at different breathing frequencies and modes for an aerosol concentration of 1.5 mg m^−3^, determined using MPPD.

MWCNT	Breathing Mode and Frequency	Thoracic Mass Deposition (µg)	Pulmonary Mass Deposition (µg)	Pulmonary Surface Area Deposition (cm^2^)
pMWCNT AR = 30 *ρ*_eff_ = 0.032 g/cm^3^	Nose-only—166/min ^$^	91.5 (0.8) ^+^	5.5 (0.9) ^+^	1.0
	**Whole-body—116.4/min ^$,^***	**109.2 (1.0)** ^+^	**6.1 (1.0)** ^+^	**1.1**
	Whole-body—93.12/min ^%^	115.0 (1.1) ^+^	6.1 (1.0) ^+^	1.1
MWCNT-COOH AR = 4 *ρ*_eff_ = 0.071 g/cm^3^	Nose-only—166/min ^$^	69.7 (0.7) ^+^	26.0 (0.6) ^+^	268.2
	**Whole-body—116.4/min ^$^***	**101.5 (1.0)** ^+^	**41.5 (1.0)** ^+^	**207.6**
	Whole-body—93.12/min ^%^	122.2 (1.2) ^+^	53.6 (1.3) ^+^	129.8

^$^ default breathing rate for breathing mode; * base case assumptions in bold text (Table 2), ^%^ default breathing rate × 80%, ^+^ ratio of variant to base case.

## References

[1] Pang J., Bachmatiuk A., Yang F., Liu H., Zhou W., Rümmeli M.H., Cuniberti G. (2021). Applications of Carbon Nanotubes in the Internet of Things Era. Nano-Micro Lett..

[2] Simon J., Flahaut E., Golzio M. (2019). Overview of Carbon Nanotubes for Biomedical Applications. Materials.

[3] Zhang C., Wu L., de Perrot M., Zhao X. (2021). Carbon Nanotubes: A Summary of Beneficial and Dangerous Aspects of an Increasingly Popular Group of Nanomaterials. Front. Oncol..

[4] Oberdörster G., Castranova V., Asgharian B., Sayre P. (2015). Inhalation Exposure to Carbon Nanotubes (CNT) and Carbon Nanofibers (CNF): Methodology and Dosimetry. J. Toxicol. Environ. Health B Crit. Rev..

[5] Kobayashi N., Izumi H., Morimoto Y. (2017). Review of toxicity studies of carbon nanotubes. J. Occup. Health.

[6] Pacurari M., Lowe K., Tchounwou P.B., Kafoury R. (2016). A Review on the Respiratory System Toxicity of Carbon Nanoparticles. Int. J. Environ. Res. Public Health.

[7] Hojo M., Maeno A., Sakamoto Y., Ohnuki A., Tada Y., Yamamoto Y., Ikushima K., Inaba R., Suzuki J., Taquahashi Y. (2022). Two-year intermittent exposure of a multiwalled carbon nanotube by intratracheal instillation induces lung tumors and pleural mesotheliomas in F344 rats. Part. Fibre Toxicol..

[8] Mitchell L.A., Gao J., Wal R.V., Gigliotti A., Burchiel S.W., McDonald J.D. (2007). Pulmonary and systemic immune response to inhaled multiwalled carbon nanotubes. Toxicol. Sci..

[9] Morimoto Y., Hirohashi M., Kobayashi N., Ogami A., Horie M., Oyabu T., Myojo T., Hashiba M., Mizuguchi Y., Kambara T. (2012). Pulmonary toxicity of well-dispersed single-wall carbon nanotubes after inhalation. Nanotoxicology.

[10] van Berlo D., Wilhelmi V., Boots A.W., Hullmann M., Kuhlbusch T.A.J., Bast A., Schins R.P.F., Albrecht C. (2014). Apoptotic, inflammatory, and fibrogenic effects of two different types of multi-walled carbon nanotubes in mouse lung. Arch. Toxicol..

[11] Chen T., Nie H., Gao X., Yang J., Pu J., Chen Z., Cui X., Wang Y., Wang H., Jia G. (2014). Epithelial-mesenchymal transition involved in pulmonary fibrosis induced by multi-walled carbon nanotubes via TGF-beta/Smad signaling pathway. Toxicol. Lett..

[12] Wang P., Nie X., Wang Y., Li Y., Ge C., Zhang L., Wang L., Bai R., Chen Z., Zhao Y. (2013). Multiwall Carbon Nanotubes Mediate Macrophage Activation and Promote Pulmonary Fibrosis Through TGF-β/Smad Signaling Pathway. Small.

[13] Vietti G., Ibouraadaten S., Palmai-Pallag M., Yakoub Y., Bailly C., Fenoglio I., Marbaix E., Lison D., van den Brule S. (2013). Towards predicting the lung fibrogenic activity of nanomaterials: Experimental validation of an in vitro fibroblast proliferation assay. Part. Fibre Toxicol..

[14] Xu J., Alexander D.B., Futakuchi M., Numano T., Fukamachi K., Suzui M., Omori T., Kanno J., Hirose A., Tsuda H. (2014). Size- and shape-dependent pleural translocation, deposition, fibrogenesis, and mesothelial proliferation by multiwalled carbon nanotubes. Cancer Sci..

[15] Saleh D.M., Alexander W.T., Numano T., Ahmed O.H.M., Gunasekaran S., Alexander D.B., Abdelgied M., El-Gazzar A.M., Takase H., Xu J. (2020). Comparative carcinogenicity study of a thick, straight-type and a thin, tangled-type multi-walled carbon nanotube administered by intra-tracheal instillation in the rat. Part. Fibre Toxicol..

[16] Fraser K., Kodali V., Yanamala N., Birch M.E., Cena L., Casuccio G., Bunker K., Lersch T.L., Evans D.E., Stefaniak A. (2020). Physicochemical characterization and genotoxicity of the broad class of carbon nanotubes and nanofibers used or produced in U.S. facilities. Part. Fibre Toxicol..

[17] Poulsen S.S., Jackson P., Kling K., Knudsen K.B., Skaug V., Kyjovska Z.O., Thomsen B.L., Clausen P.A., Atluri R., Berthing T. (2016). Multi-walled carbon nanotube physicochemical properties predict pulmonary inflammation and genotoxicity. Nanotoxicology.

[18] Knudsen K.B., Berthing T., Jackson P., Poulsen S.S., Mortensen A., Jacobsen N.R., Skaug V., Szarek J., Hougaard K.S., Wolff H. (2019). Physicochemical predictors of Multi-Walled Carbon Nanotube–induced pulmonary histopathology and toxicity one year after pulmonary deposition of 11 different Multi-Walled Carbon Nanotubes in mice. Basic Clin. Pharmacol. Toxicol..

[19] Hadrup N., Knudsen K.B., Carriere M., Mayne-L’Hermite M., Bobyk L., Allard S., Miserque F., Pibaleau B., Pinault M., Wallin H. (2021). Safe-by-design strategies for lowering the genotoxicity and pulmonary inflammation of multiwalled carbon nanotubes: Reduction of length and the introduction of COOH groups. Environ. Toxicol. Pharmacol..

[20] Solorio-Rodriguez S.A., Williams A., Poulsen S.S., Knudsen K.B., Jensen K.A., Clausen P.A., Danielsen P.H., Wallin H., Vogel U., Halappanavar S. (2023). Single-Walled vs. Multi-Walled Carbon Nanotubes: Influence of Physico-Chemical Properties on Toxicogenomics Responses in Mouse Lungs. Nanomaterials.

[21] Danielsen P.H., Poulsen S.S., Knudsen K.B., Clausen P.A., Jensen K.A., Wallin H., Vogel U. (2024). Physicochemical properties of 26 carbon nanotubes as predictors for pulmonary inflammation and acute phase response in mice following intratracheal lung exposure. Environ. Toxicol. Pharmacol..

[22] Gaté L., Knudsen K.B., Seidel C., Berthing T., Chézeau L., Jacobsen N.R., Valentino S., Wallin H., Bau S., Wolff H. (2019). Pulmonary toxicity of two different multi-walled carbon nanotubes in rat: Comparison between intratracheal instillation and inhalation exposure. Toxicol. Appl. Pharmacol..

[23] Seidel C., Zhernovkov V., Cassidy H., Kholodenko B., Matallanas D., Cosnier F., Gaté L. (2021). Inhaled multi-walled carbon nanotubes differently modulate global gene and protein expression in rat lungs. Nanotoxicology.

[24] Donaldson K., Murphy F.A., Duffin R., Poland C.A. (2010). Asbestos, carbon nanotubes and the pleural mesothelium: A review of the hypothesis regarding the role of long fibre retention in the parietal pleura, inflammation and mesothelioma. Part. Fibre Toxicol..

[25] Nagai H., Okazaki Y., Chew S.H., Misawa N., Yamashita Y., Akatsuka S., Ishihara T., Yamashita K., Yoshikawa Y., Yasui H. (2011). Diameter and rigidity of multiwalled carbon nanotubes are critical factors in mesothelial injury and carcinogenesis. Proc. Natl. Acad. Sci. USA.

[26] Murphy F., Dekkers S., Braakhuis H., Ma-Hock L., Johnston H., Janer G., di Cristo L., Sabella S., Jacobsen N.R., Oomen A.G. (2021). An integrated approach to testing and assessment of high aspect ratio nanomaterials and its application for grouping based on a common mesothelioma hazard. NanoImpact.

[27] Murphy F., Jacobsen N.R., Di Ianni E., Johnston H., Braakhuis H., Peijnenburg W., Oomen A., Fernandes T., Stone V. (2022). Grouping MWCNTs based on their similar potential to cause pulmonary hazard after inhalation: A case-study. Part. Fibre Toxicol..

[28] Asgharian B., Anjilvel S. (1998). A multiple-path model of fiber deposition in the rat lung. Toxicol. Sci..

[29] Rasmussen K., Mast J., De Temmerman P., Verleysen E., Waegeneers N., Van Steen F., Pizzolon J., De Temmerman L., Van Doren E., Joint Research Centre: Institute for Health and Consumer Protection (2014). Multi-Walled Carbon Nanotubes, NM-400, NM-401, NM-402, NM-403, Characterisation and Physico-Chemical Properties.

[30] McKinney W., Chen B., Frazer D. (2009). Computer controlled multi-walled carbon nanotube inhalation exposure system. Inhal. Toxicol..

[31] Wright M.D., Buckley A.J., Smith R. (2020). Estimates of carbon nanotube deposition in the lung: Improving quality and robustness. Inhal. Toxicol..

[32] Landsiedel R., Ma-Hock L., Hofmann T., Wiemann M., Strauss V., Treumann S., Wohlleben W., Gröters S., Wiench K., van Ravenzwaay B. (2014). Application of short-term inhalation studies to assess the inhalation toxicity of nanomaterials. Part. Fibre Toxicol..

[33] Piao Y., Liu Y., Xie X. (2013). Change trends of organ weight background data in sprague dawley rats at different ages. J. Toxicol. Pathol..

[34] Song J.A., Yang H.S., Lee J., Kwon S., Jung K.J., Heo J.D., Cho K.H., Song C.W., Lee K. (2010). Standardization of bronchoalveolar lavage method based on suction frequency number and lavage fraction number using rats. Toxicol. Res..

[35] Dong J., Ma Q. (2017). Osteopontin enhances multi-walled carbon nanotube-triggered lung fibrosis by promoting TGF-β1 activation and myofibroblast differentiation. Part. Fibre Toxicol..

[36] Han L., Haefner V., Yildirim A., Adler H., Stoeger T. (2025). Carbonaceous particle exposure triggered accumulation of Osteopontin/SPP1+ macrophages contributes to emphysema development. MedComm.

[37] Morimoto Y., Hirohashi M., Ogami A., Oyabu T., Myojo T., Todoroki M., Yamamoto M., Hashiba M., Mizuguchi Y., Lee B.W. (2012). Pulmonary toxicity of well-dispersed multi-wall carbon nanotubes following inhalation and intratracheal instillation. Nanotoxicology.

[38] Kim J.S., Sung J.H., Choi B.G., Ryu H.Y., Song K.S., Shin J.H., Lee J.S., Hwang J.H., Lee J.H., Lee G.H. (2014). In vivo genotoxicity evaluation of lung cells from Fischer 344 rats following 28 days of inhalation exposure to MWCNTs, plus 28 days and 90 days post-exposure. Inhal. Toxicol..

[39] Kim J.K., Jo M.S., Kim Y., Kim T.G., Shin J.H., Kim B.W., Kim H.P., Lee H.K., Kim H.S., Ahn K. (2020). 28-Day inhalation toxicity study with evaluation of lung deposition and retention of tangled multi-walled carbon nanotubes. Nanotoxicology.

[40] Kasai T., Umeda Y., Ohnishi M., Kondo H., Takeuchi T., Aiso S., Nishizawa T., Matsumoto M., Fukushima S. (2015). Thirteen-week study of toxicity of fiber-like multi-walled carbon nanotubes with whole-body inhalation exposure in rats. Nanotoxicology.

[41] Ma-Hock L., Treumann S., Strauss V., Brill S., Luizi F., Mertler M., Wiench K., Gamer A.O., van Ravenzwaay B., Landsiedel R. (2009). Inhalation toxicity of multiwall carbon nanotubes in rats exposed for 3 months. Toxicol. Sci..

[42] Pauluhn J. (2010). Subchronic 13-week inhalation exposure of rats to multiwalled carbon nanotubes: Toxic effects are determined by density of agglomerate structures, not fibrillar structures. Toxicol. Sci..

[43] OECD (2018). Test No. 412: Subacute Inhalation Toxicity: 28-Day Study. OECD Guidelines for the Testing of Chemicals, Section 4.

[44] OECD (2018). Test No. 413: Subchronic Inhalation Toxicity: 90-day Study. OECD Guidelines for the Testing of Chemicals, Section 4.

[45] Devoy J., Al-Abed S., Cerdan B., Cho W.S., Dubuc D., Flahaut E., Grenier K., Grossmann S., Gulumian M., Jeong J. (2024). Analysis of carbon nanotube levels in organic matter: An inter-laboratory comparison to determine best practice. Nanotoxicology.

[46] Petersen E.J., Flores-Cervantes D.X., Bucheli T.D., Elliott L.C.C., Fagan J.A., Gogos A., Hanna S., Kägi R., Mansfield E., Bustos A.R.M. (2016). Quantification of Carbon Nanotubes in Environmental Matrices: Current Capabilities, Case Studies, and Future Prospects. Environ. Sci. Technol..

[47] Schmid O., Stoeger T. (2016). Surface area is the biologically most effective dose metric for acute nanoparticle toxicity in the lung. J. Aerosol Sci..

[48] Cosnier F., Seidel C., Valentino S., Schmid O., Bau S., Vogel U., Devoy J., Gaté L. (2021). Retained particle surface area dose drives inflammation in rat lungs following acute, subacute, and subchronic inhalation of nanomaterials. Part. Fibre Toxicol..

[49] Li R., Wang X., Ji Z., Sun B., Zhang H., Chang C.H., Lin S., Meng H., Liao Y.-P., Wang M. (2013). Surface Charge and Cellular Processing of Covalently Functionalized Multiwall Carbon Nanotubes Determine Pulmonary Toxicity. ACS Nano.

[50] Sager T.M., Wolfarth M.W., Andrew M., Hubbs A., Friend S., Chen T.H., Porter D.W., Wu N., Yang F., Hamilton R.F. (2014). Effect of multi-walled carbon nanotube surface modification on bioactivity in the C57BL/6 mouse model. Nanotoxicology.

[51] Duke K.S., Taylor-Just A.J., Ihrie M.D., Shipkowski K.A., Thompson E.A., Dandley E.C., Parsons G.N., Bonner J.C. (2017). STAT1-dependent and -independent pulmonary allergic and fibrogenic responses in mice after exposure to tangled versus rod-like multi-walled carbon nanotubes. Part. Fibre Toxicol..

[52] Rydman E.M., Ilves M., Vanhala E., Vippola M., Lehto M., Kinaret P.A., Pylkkänen L., Happo M., Hirvonen M.R., Greco D. (2015). A Single Aspiration of Rod-like Carbon Nanotubes Induces Asbestos-like Pulmonary Inflammation Mediated in Part by the IL-1 Receptor. Toxicol. Sci..

[53] DeCarlo P.F., Slowik J.G., Worsnop D.R., Davidovits P., Jimenez J.L. (2004). Particle Morphology and Density Characterization by Combined Mobility and Aerodynamic Diameter Measurements. Part 1: Theory. Aerosol Sci. Technol..

[54] Ku B.K., Kulkarni P. (2015). Measurement of Transport Properties of Aerosolized Nanomaterials. J. Aerosol Sci..

[55] Wang J., Bahk Y.K., Chen S.-C., Pui D.Y.H. (2015). Characteristics of airborne fractal-like agglomerates of carbon nanotubes. Carbon.

[56] Ménache M.G., Miller F.J., Raabe O.G. (1995). Particle inhalability curves for humans and small laboratory animals. Ann. Occup. Hyg..

[57] Miller F.J., Asgharian B., Schroeter J.D., Price O., Corley R.A., Einstein D.R., Jacob R.E., Cox T.C., Kabilan S., Bentley T. (2014). Respiratory tract lung geometry and dosimetry model for male Sprague-Dawley rats. Inhal. Toxicol..

[58] Oyabu T., Morimoto Y., Izumi H., Yoshiura Y., Tomonaga T., Lee B.W., Okada T., Myojo T., Shimada M., Kubo M. (2016). Comparison between whole-body inhalation and nose-only inhalation on the deposition and health effects of nanoparticles. Environ. Health Prev. Med..

[59] Yeh H.C., Snipes M.B., Eidson A.F., Hobbs C.H., Henry M.C. (1990). Comparative Evaluation of Nose-Only Versus Whole-Body Inhalation Exposures for Rats—Aerosol Characteristics and Lung Deposition. Inhal. Toxicol..

[60] Jagiello K., Halappanavar S., Rybińska-Fryca A., Willliams A., Vogel U., Puzyn T. (2021). Transcriptomics-Based and AOP-Informed Structure-Activity Relationships to Predict Pulmonary Pathology Induced by Multiwalled Carbon Nanotubes. Small.

[61] Merugu S., Jagiello K., Gajewicz-Skretna A., Halappanavar S., Willliams A., Vogel U., Puzyn T. (2025). The Impact of Carbon Nanotube Properties on Lung Pathologies and Atherosclerosis Through Acute Inflammation: A New AOP-Anchored in Silico NAM. Small.

[62] Sager T.M., Umbright C.M., Mustafa G.M., Roberts J.R., Orandle M.S., Cumpston J.L., McKinney W.G., Boots T., Kashon M.L., Joseph P. (2022). Pulmonary toxicity and gene expression changes in response to whole-body inhalation exposure to multi-walled carbon nanotubes in rats. Inhal. Toxicol..

[63] Fujita K., Fukuda M., Endoh S., Maru J., Kato H., Nakamura A., Shinohara N., Uchino K., Honda K. (2015). Size effects of single-walled carbon nanotubes on in vivo and in vitro pulmonary toxicity. Inhal. Toxicol..

[64] Francis A.P., Ganapathy S., Palla V.R., Murthy P.B., Ramaprabhu S., Devasena T. (2015). One time nose-only inhalation of MWCNTs: Exploring the mechanism of toxicity by intermittent sacrifice in Wistar rats. Toxicol. Rep..

[65] Mercer R.R., Scabilloni J.F., Hubbs A.F., Battelli L.A., McKinney W., Friend S., Wolfarth M.G., Andrew M., Castranova V., Porter D.W. (2013). Distribution and fibrotic response following inhalation exposure to multi-walled carbon nanotubes. Part. Fibre Toxicol..

[66] Porter D.W., Hubbs A.F., Chen B.T., McKinney W., Mercer R.R., Wolfarth M.G., Battelli L., Wu N., Sriram K., Leonard S. (2013). Acute pulmonary dose-responses to inhaled multi-walled carbon nanotubes. Nanotoxicology.

[67] Ryman-Rasmussen J.P., Cesta M.F., Brody A.R., Shipley-Phillips J.K., Everitt J.I., Tewksbury E.W., Moss O.R., Wong B.A., Dodd D.E., Andersen M.E. (2009). Inhaled carbon nanotubes reach the subpleural tissue in mice. Nat. Nanotechnol..

[68] Ryman-Rasmussen J.P., Tewksbury E.W., Moss O.R., Cesta M.F., Wong B.A., Bonner J.C. (2009). Inhaled multiwalled carbon nanotubes potentiate airway fibrosis in murine allergic asthma. Am. J. Respir. Cell. Mol. Biol..

[69] Shvedova A.A., Kisin E., Murray A.R., Johnson V.J., Gorelik O., Arepalli S., Hubbs A.F., Mercer R.R., Keohavong P., Sussman N. (2008). Inhalation vs. aspiration of single-walled carbon nanotubes in C57BL/6 mice: Inflammation, fibrosis, oxidative stress, and mutagenesis. Am. J. Physiol. Lung. Cell. Mol. Physiol..

[70] Shvedova A.A., Yanamala N., Kisin E.R., Tkach A.V., Murray A.R., Hubbs A., Chirila M.M., Keohavong P., Sycheva L.P., Kagan V.E. (2014). Long-term effects of carbon containing engineered nanomaterials and asbestos in the lung: One year postexposure comparisons. Am. J. Physiol. Lung. Cell. Mol. Physiol..

[71] Treumann S., Ma-Hock L., Gröters S., Landsiedel R., van Ravenzwaay B. (2013). Additional histopathologic examination of the lungs from a 3-month inhalation toxicity study with multiwall carbon nanotubes in rats. Toxicol. Sci..

[72] Dong J., Ma Q. (2018). Type 2 Immune Mechanisms in Carbon Nanotube-Induced Lung Fibrosis. Front. Immunol..

[73] Khaliullin T.O., Kisin E.R., Murray A.R., Yanamala N., Shurin M.R., Gutkin D.W., Fatkhutdinova L.M., Kagan V.E., Shvedova A.A. (2017). Mediation of the single-walled carbon nanotubes induced pulmonary fibrogenic response by osteopontin and TGF-β1. Exp. Lung Res..

[74] Dong J., Ma Q. (2017). TIMP1 promotes multi-walled carbon nanotube-induced lung fibrosis by stimulating fibroblast activation and proliferation. Nanotoxicology.

[75] Guo C., Buckley A., Marczylo T., Seiffert J., Römer I., Warren J., Hodgson A., Chung K.F., Gant T.W., Smith R. (2018). The small airway epithelium as a target for the adverse pulmonary effects of silver nanoparticle inhalation. Nanotoxicology.

[76] Guo C., Robertson S., Weber R.J.M., Buckley A., Warren J., Hodgson A., Rappoport J.Z., Ignatyev K., Meldrum K., Römer I. (2019). Pulmonary toxicity of inhaled nano-sized cerium oxide aerosols in Sprague-Dawley rats. Nanotoxicology.

[77] Bermudez E., Mangum J.B., Wong B.A., Asgharian B., Hext P.M., Warheit D.B., Everitt J.I. (2004). Pulmonary responses of mice, rats, and hamsters to subchronic inhalation of ultrafine titanium dioxide particles. Toxicol. Sci..

[78] Elder A., Gelein R., Finkelstein J.N., Driscoll K.E., Harkema J., Oberdörster G. (2005). Effects of subchronically inhaled carbon black in three species. I. Retention kinetics, lung inflammation, and histopathology. Toxicol. Sci..

[79] Keller J., Wohlleben W., Ma-Hock L., Strauss V., Gröters S., Küttler K., Wiench K., Herden C., Oberdörster G., van Ravenzwaay B. (2014). Time course of lung retention and toxicity of inhaled particles: Short-term exposure to nano-Ceria. Arch. Toxicol..

[80] Ellinger-Ziegelbauer H., Pauluhn J. (2009). Pulmonary toxicity of multi-walled carbon nanotubes (Baytubes^®^) relative to α-quartz following a single 6h inhalation exposure of rats and a 3 months post-exposure period. Toxicology.

[81] Kuempel E.D., O’Flaherty E.J., Stayner L.T., Smith R.J., Green F.H., Vallyathan V. (2001). A biomathematical model of particle clearance and retention in the lungs of coal miners. Regul. Toxicol. Pharmacol..

[82] Gregoratto D., Bailey M.R., Marsh J.W. (2010). Modelling particle retention in the alveolar-interstitial region of the human lungs. J. Radiol. Prot..

[83] Debia M., Bakhiyi B., Ostiguy C., Verbeek J.H., Brouwer D.H., Murashov V. (2016). A Systematic Review of Reported Exposure to Engineered Nanomaterials. Ann. Occup. Hyg..

[84] Dahm M.M., Schubauer-Berigan M.K., Evans D.E., Birch M.E., Bertke S., Beard J.D., Erdely A., Fernback J.E., Mercer R.R., Grinshpun S.A. (2018). Exposure assessments for a cross-sectional epidemiologic study of US carbon nanotube and nanofiber workers. Int. J. Hyg. Environ. Health.

[85] NIOSH (2013). Current Intelligence Bulletin 65: Occupational Exposure to Carbon Nanotubes and Nanofibers.

[86] Guseva Canu I., Bateson T.F., Bouvard V., Debia M., Dion C., Savolainen K., Yu I.J. (2016). Human exposure to carbon-based fibrous nanomaterials: A review. Int. J. Hyg. Environ. Health.

[87] Ku B.K., Birch M.E. (2019). Aerosolization and characterization of carbon nanotube and nanofiber materials: Relationship between aerosol properties and bulk density. J. Aerosol Sci..

[88] Carley D.W., Berecek K., Videnovic A., Radulovacki M. (2000). Sleep-disordered respiration in phenotypically normotensive, genetically hypertensive rats. Am. J. Respir. Crit. Care Med..

[89] Stephenson R., Liao K.S., Hamrahi H., Horner R.L. (2001). Circadian rhythms and sleep have additive effects on respiration in the rat. J. Physiol..

