# Supplementary material for: Pulmonary Toxicity of Long, Thick MWCNT and Very Long, Thin Carboxylated MWCNT Aerosols Following 28 Days Whole-Body Exposure
Source: Toxics. 2025 May 16;13(5):401. doi: 10.3390/toxics13050401 (PMC12115612; doi:10.3390/toxics13050401)
Supplement: Supplementary file 1 [file toxics-13-00401-s001.zip › toxics-3578255-supplementary.pdf]

Supplementary Information

Pulmonary toxicity of long, thick MWCNT and very long, thin carboxylated MWCNT aerosols following 28 days whole-body exposure

Chang Guo [1,*], Matthew D. Wright [1], Alison Buckley [1], Adam Laycock [1], Trine Berthing [2], Ulla Vogel [2], Frédéric Cosnier [3], Laurent Gaté [3], Martin O. Leonard [1] and Rachel Smith [1,*]

1 Toxicology Department, UK Health Security Agency, Harwell Campus, Didcot, OX11 0RQ, UK; chang.guo@ukhsa.gov.uk (C.G.); matthew.d.wright@ukhsa.gov.uk (M.W.); alison.buckley@ukhsa.gov.uk (A.B.); adam.laycock@ukhsa.gov.uk (A.L.); martin.leonard@ukhsa.gov.uk (M.L.); rachel.smith@ukhsa.gov.uk (R.S.)

2 National Research Centre for the Working Environment, DK-2100 Copenhagen, Denmark; trb@nfa.dk (T.B.); ubv@nfa.dk (U.V.)

3 French Research and Safety Institute for the Prevention of Occupational Accidents and Diseases (INRS), Toxicology and Biomonitoring Division, 54519 Vandoeuvre les Nancy, France; frederic.cosnier@inrs.fr (F.C.); laurent.gate@inrs.fr (L.G.)

* Correspondence: chang.guo@ukhsa.gov.uk; rachel.smith@ukhsa.gov.uk

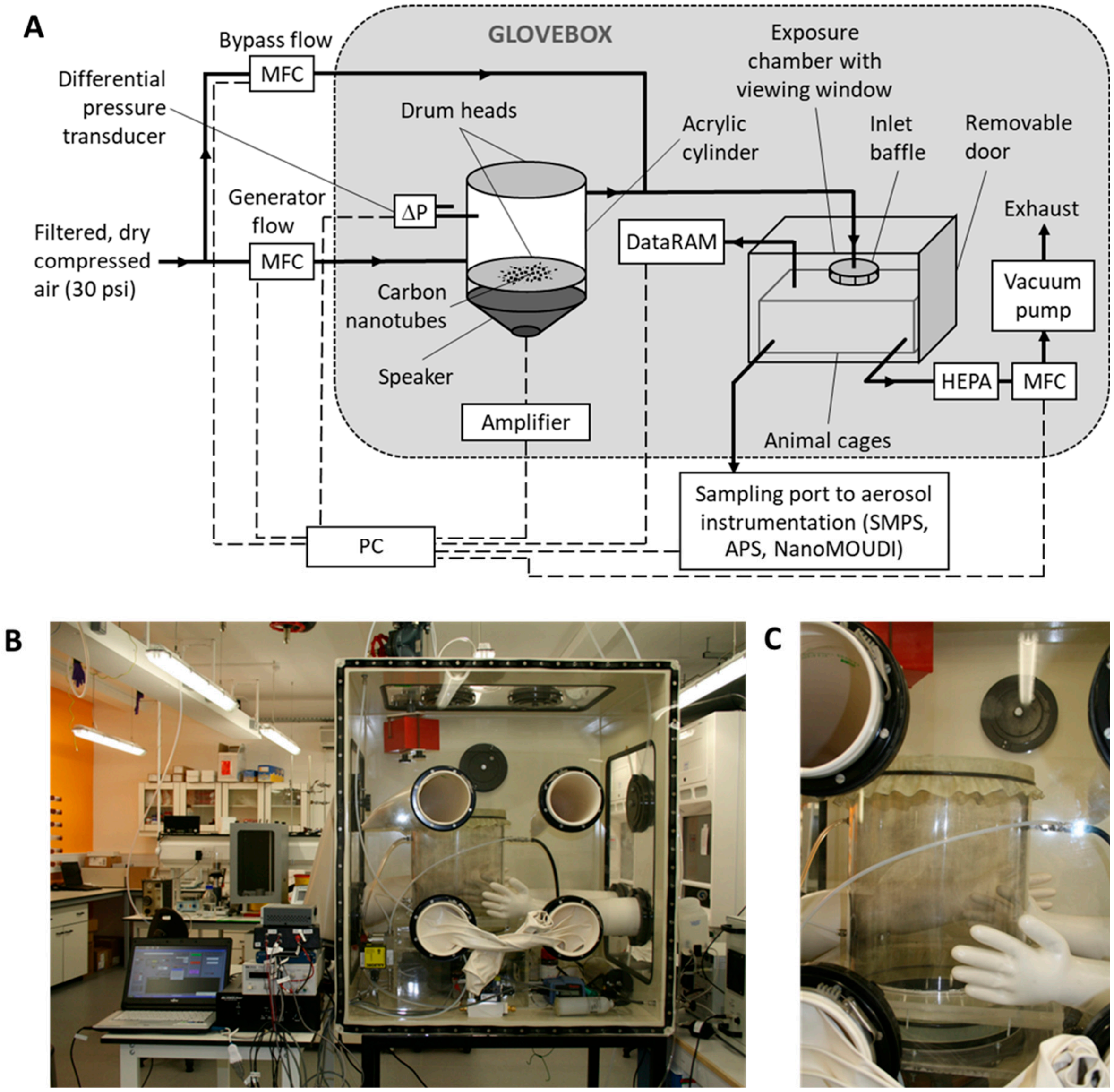

Figure S1. Experimental aerosol exposure set-up: (**A**) schematic diagram of the experimental set-up (MFC: mass flow controller, HEPA: high-efficiency particulate air filter, SMPS: scanning mobility particle sizer), (**B**) and (**C**) photographs of aerosol generator.

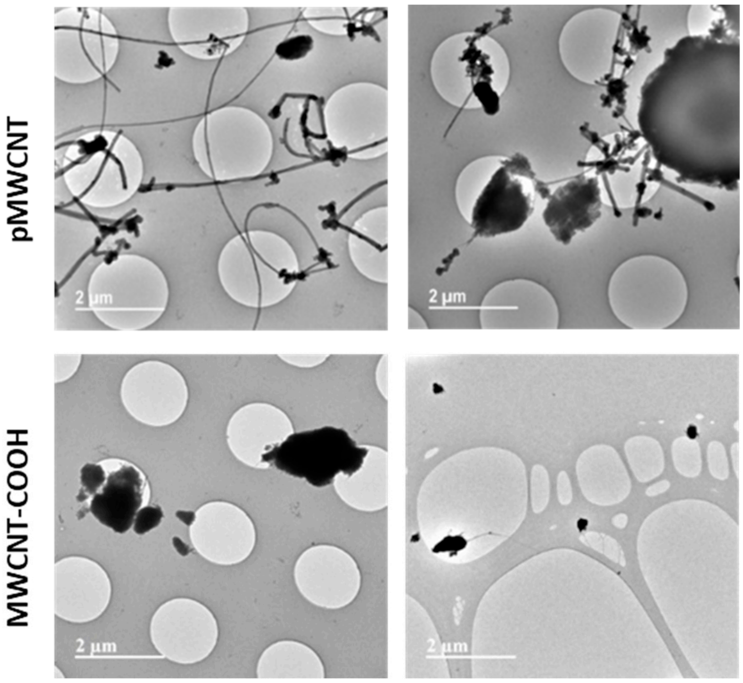

Figure S2. Representative TEM images of MWCNT aerosol particles.

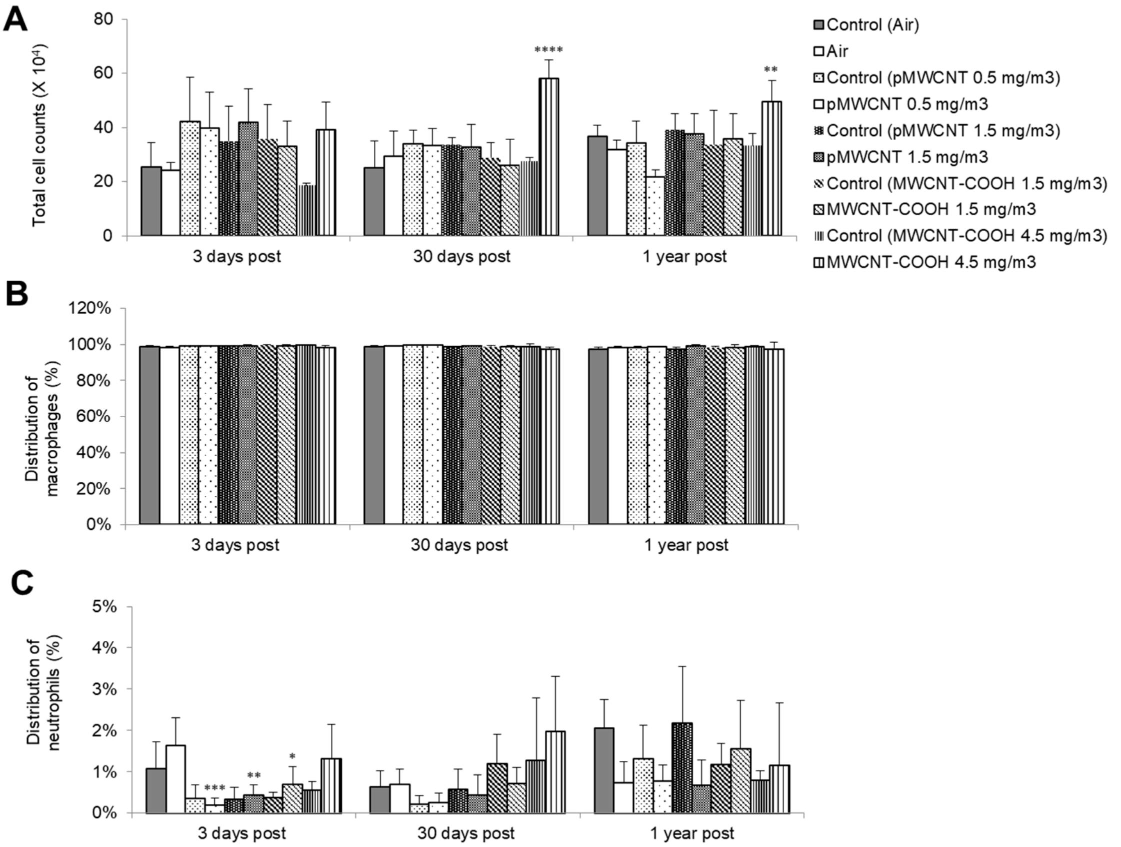

Figure S3. Cytological analysis of bronchoalveolar lavage fluid: (**A**) Total cell counts; (B) Distribution of macrophages; (C) Distribution of neutrophils. Values expressed as mean +/- SD. Significance was determined by one-way ANOVA versus air-exposed groups with Dunnett's post-test: *, $p < 0.05$, **, $p < 0.01$, ***, $p < 0.001$, and ****, $p < 0.0001$.

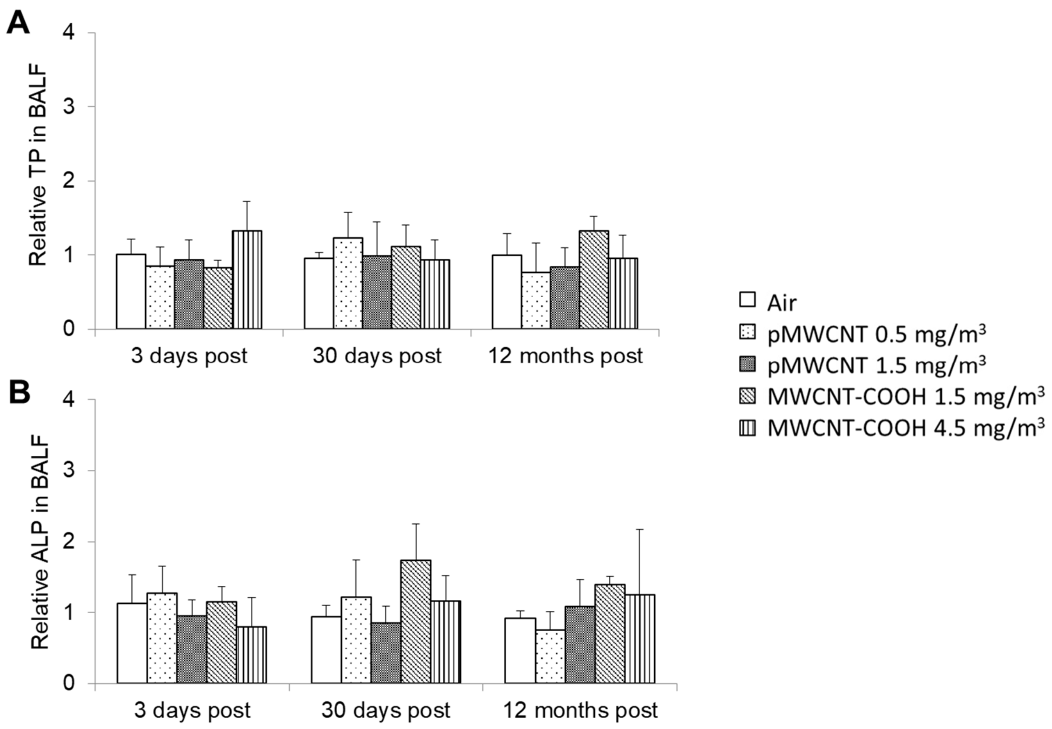

Figure S4. Cytotoxicity analysis of bronchoalveolar lavage fluid. (A) Relative total protein (TP) levels ; (B) Relative alkaline phosphatase (ALP) levels. Results are normalised to distinct unexposed control group. No significance was observed in comparison to distinct unexposed control groups.

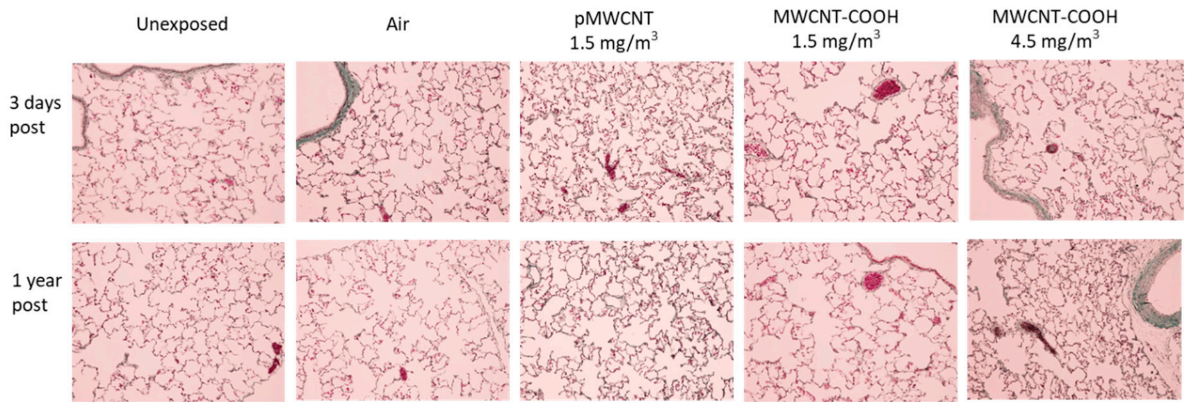

Figure S5. Trichrome-Masson stained lung sections from rats exposed to two types of MWCNT aerosols at 3 days and 1 year post-exposure.

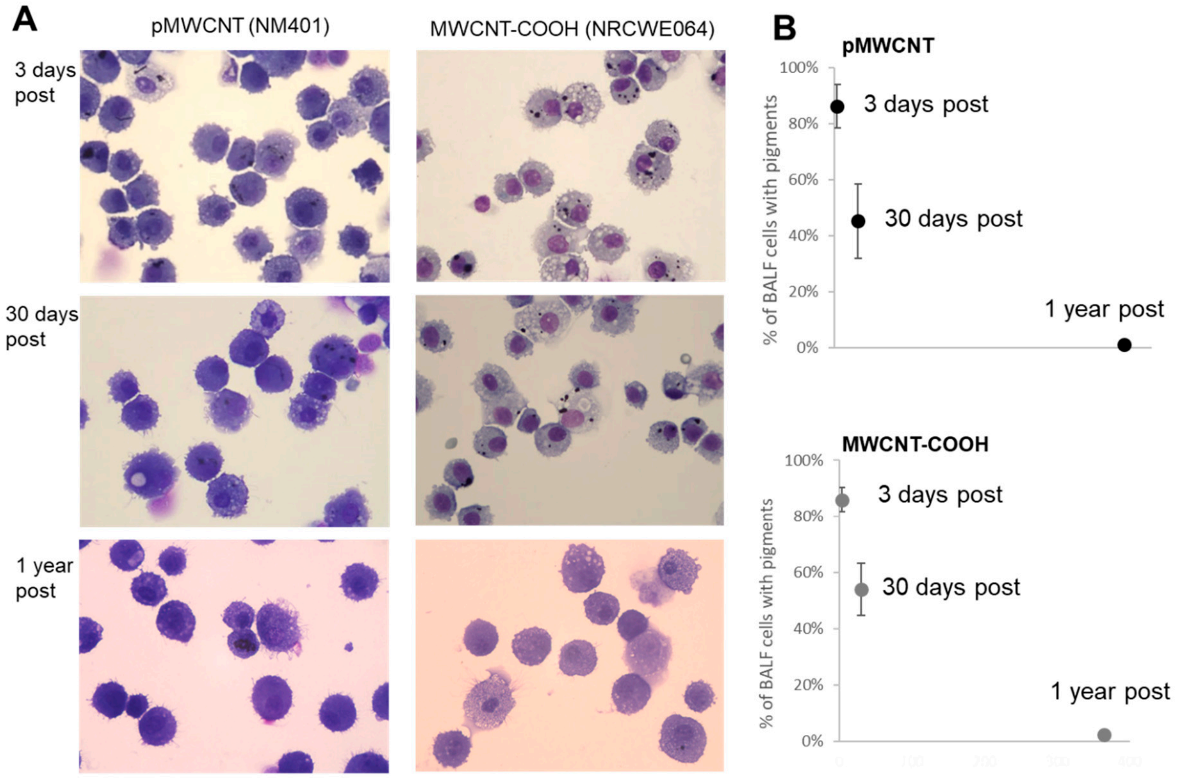

Figure S6. Localisation of inhaled MWCNT particles in macrophages recovered by bronchoalveolar lavage from rats at 3 days, 30 days, and 1 year post-exposure to two types of MWCNT aerosols at the medium aerosol concentration (1.5 mg/m^3): (**A**) representative brightfield microscope images of recovered macrophages and (**B**) percentage of macrophages with observed pigments (agglomerates of MWCNTs) recovered from bronchoalveolar lavage. The error bars for groups for 1 year post-exposure are too small to be clearly observed.

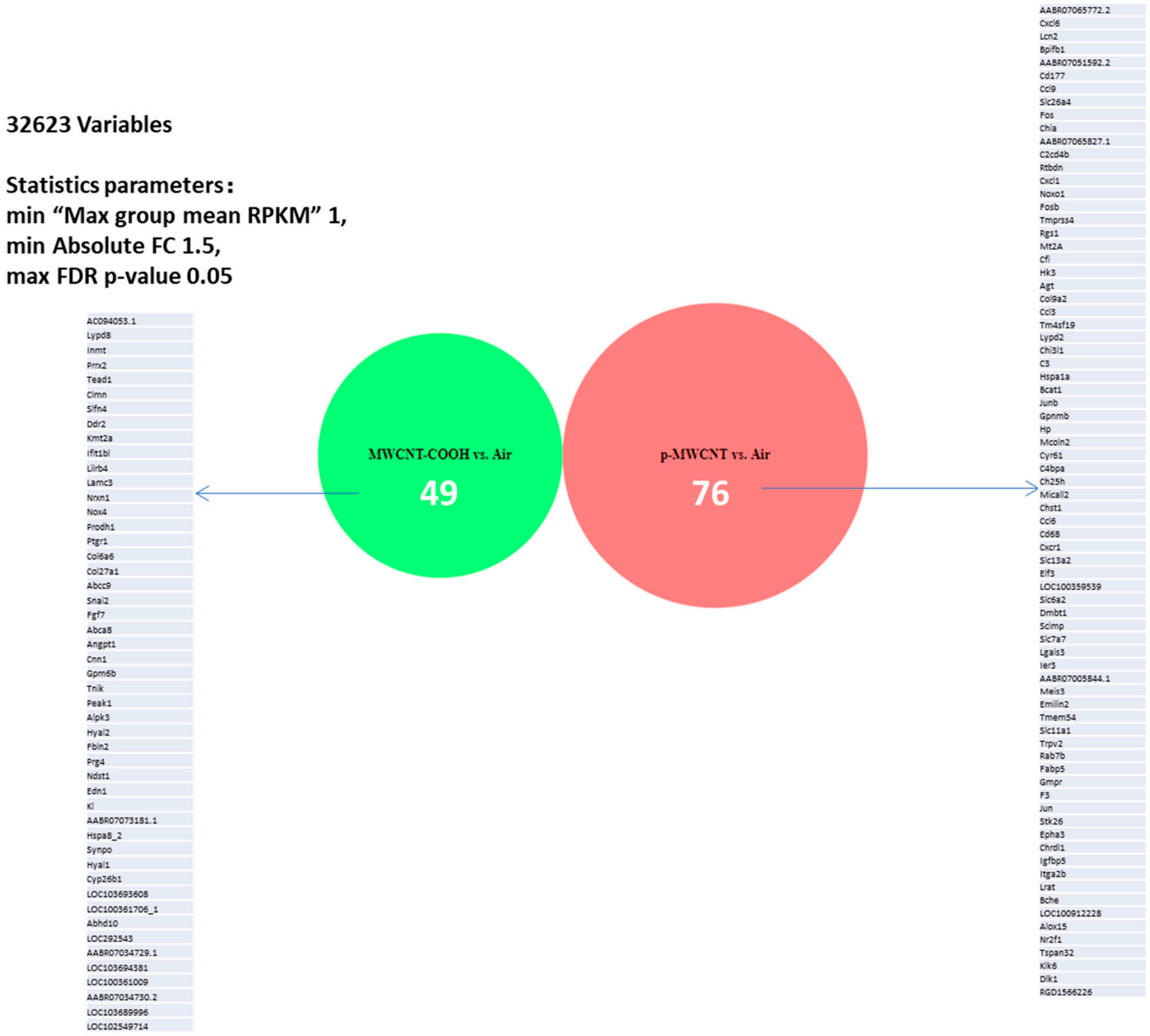

Figure S7. Venn diagram of significant differentially expressed genes (DEGs) compared between two types of MWCNT aerosols, with significant differential expression in lung tissues from rats exposed to MWCNT-COOH or pMWCNT (NM401) at 3 days post-exposure.

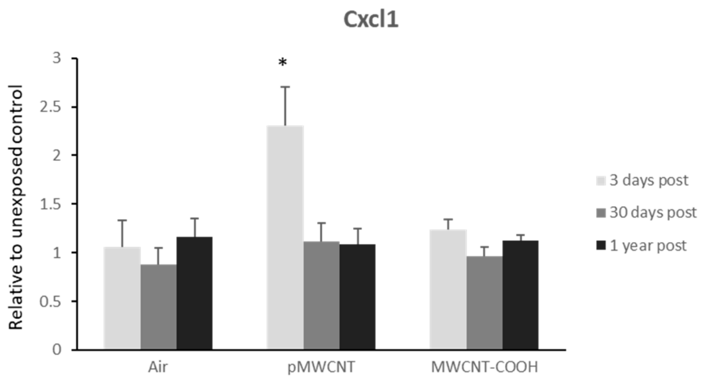

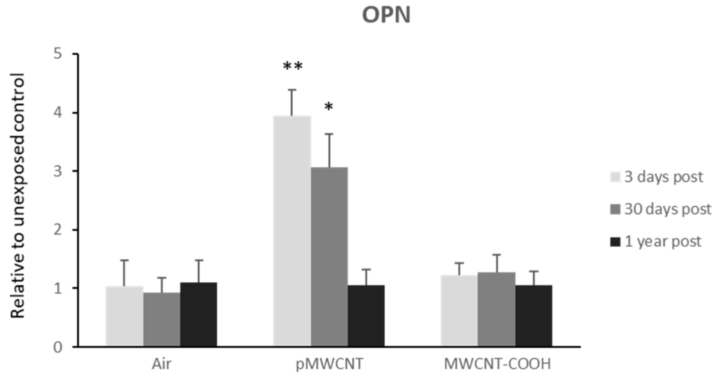

Figure S8. Expression of cytokines Cxcl1 and OPN, in protein levels in BALF from rats at 3 days, 30 days, and 1 year post-exposure to the medium aerosol concentration of two types of MWCNT aerosols relative to the unexposed controls at different time points post-exposure. A p-value of less than 0.05 was considered statistically significant: *, $p < 0.05$; **, and $p < 0.01$.

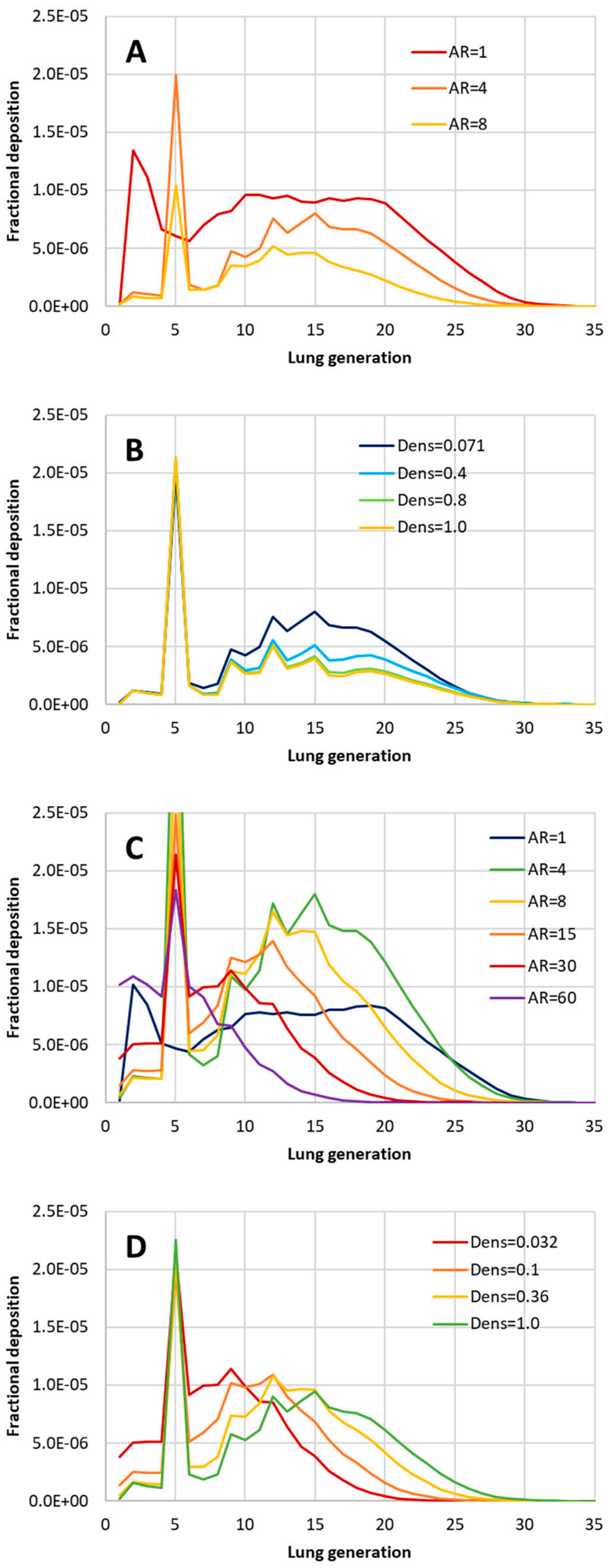

Figure S9. Regional deposition pattern for each MWCNT (A and B = MWCNT-COOH, C and D = pMWCNT) for target concentration 1.5 mg m^{-3} showing the effect of altering aspect ratio (A and C) and effective density (B and D) on computed MPPD results.

Table S1. Elemental impurities detected in MWNCTs using ICP-MS.

Element	MWCNT-COOH Concentration Mean ± SD (ppm)	pMWCNT Concentration Mean ± SD (ppm)
Co*	1720 ± 240	0.614 ± 0.160
Fe*	632 ± 90	3430 ± 207
Mo*	414 ± 54	ND
Cr*	311 ± 38	10.4 ± 0.9
Mg	167 ± 28	7.95 ± 3.60
Ni*	144 ± 26	0.393 ± 0.411
K	40.1 ± 7.3	8.31 ± 3.94
Mn	22.2 ± 2.9	ND
Ti	14.2 ± 2.5	2.56 ± 0.33
Al	9.96 ± 3.07	4.20 ± 1.29
La	9.79 ± 0.88	ND
Sr	9.58 ± 1.17	ND
Na	9.03 ± 5.06	ND
V	4.17 ± 0.51	0.455 ± 0.103
Cu*	2.65 ± 0.25	0.796 ± 0.131
Ba	1.58 ± 0.21	0.241 ± 0.417
As	1.34 ± 0.20	ND
Ce	0.0519 ± 0.0045	14.7 ± 1.4

*Common trace elements associated with CNT synthesis. Only identified elements with concentrations > 1 ppm in one or both materials have been included in the table.

Analysis method: approximately 10 mg pMWCNT (n=6) and 100 mg MWCNT-COOH (n=3) were weighed directly into the microwave digestion vials. To these 3 mL HNO_3 and 3 mL H_2O_2 were added to the samples and left at room temperature for 15 minutes before placing in an Analytix Ultrawave microwave digester where the temperature was ramped to 240°C over 25 minutes and held for an additional 20 minutes. Once cooled, the samples were transferred to trace metal grade sample vials and diluted to an acid matrix of 2%. Analysis was performed using a Thermo iCAPQ ICP-MS instrument. Freshly prepared multi-element calibration standards were prepared at concentrations of 0.1, 0.5, 1, 5, 10, 50 and 100 $\mu g\ L^{-1}$ with a 1 $\mu g\ L^{-1}$ Rh solution added to all standards and samples online.

Table S2. Aerosol parameters for each mode obtained from fitting bimodal log-normal distributions to mass size distributions derived from APS results.

MWCNT	Target conc. (mg m^{-3})	Mode	MMAD (μm)	GSD	Mass fraction
pMWCNT	0.5 (Low)	1	1.256	1.644	0.695
		2	1.943	1.270	0.305
	1.5 (Medium)*	1	0.924	1.448	0.267
		2	2.035	1.388	0.554
MWCNT-COOH	1.5 (Medium)	1	1.930	1.536	0.489
		2	2.265	1.278	0.511
	4.5 (High)	1	1.857	1.560	0.459
		2	2.315	1.293	0.541

*When fitting, a best fit was observed for this distribution with 4 modes, but upon calculating deposition in MPPD, the two larger size modes contributed a negligible amount to deposition, so were neglected henceforth; however, this is the reason that the sum of the mass fractions given here is not = 1.

Table S3. Pulmonary distribution of MWCNTs from enhanced darkfield microscopy images—incidence table.

MWCNT distribution	pMWCNT		MWCNT-COOH	
	**Day 3 (n=3)**	**1 year (n=6)**	**Day 3 (n=3)**	**1 year (n=3)**
Lung samples with more than 3 MWCNT observations in a partial scan	3/3	4/6	3/3	0/3
MWCNT observed in:				
Airway	1/3	1/6	(0/3)	(0/3)
Alveolar macrophage	3/3	4/6	3/3	(2/3)
Higher density at terminal/respiratory bronchiole	0/3	0/6	0/3	0/3
Interstitial	3/3	4/6	(0/3)	(1/3)
Lymphocytic infiltration/lymphoid tissue	1/3	0/6	0/3	(0/3)
Perivascular	0/3	2/6	(0/3)	(0/3)
"Free" in Alveoli	3/3	2/6	(0/3)	(1/3)

Note: In enhanced darkfield microscopy images, the MWCNT-COOH agglomerates are similar in appearance to common artefacts in tissue samples; therefore, some incidences are uncertain, and these are in parentheses.
